# miR-183-5p-enriched extracellular vesicles promote the crosstalk between hepatocellular carcinoma cell and endothelial cell via SIK1/PI3K/AKT and CCL20/CCR6 signaling pathways

**DOI:** 10.3389/fonc.2025.1532239

**Published:** 2025-03-06

**Authors:** Ye Han, Wu-shuang Gong, Xue-sha Xing, Hang Zhou, Xiao-lei Wang, Yi Xu, Xian-li Zhou, Wei-li Xue

**Affiliations:** ^1^ In-Patient Ultrasound Department, The Second Affiliated Hospital of Harbin Medical University, Harbin, China; ^2^ Department of Hepatopancreatobiliary Surgery, The Second Affiliated Hospital of Harbin Medical University, Harbin, China; ^3^ Department of Pathology, Li Ka Shing Faculty of Medicine, The University of Hong Kong, Hong Kong, Hong Kong SAR, China

**Keywords:** hepatocellular carcinoma, extracellular vesicles, HUVECs, miR-183-5p, SIK1

## Abstract

**Background:**

The cancer-related mortality of primary liver cancer ranks third globally, and hepatocellular carcinoma (HCC) is predominant, posing a serious threat to patients’ health. Understanding HCC’s pathogenesis and target molecules is crucial for early diagnosis and prognosis. Extracellular vesicles (EVs) and their carried miRNAs impact tumor progression. This study aims to investigate miR-183-5p in HCC cell-derived EVs on angiogenesis, progression, and metastasis, and provide diagnostic and therapeutic evidence.

**Methods:**

qRT-PCR was used to evaluate the expression of miR-183-5p in HCC tissue and plasma EV samples. Contrast-enhanced ultrasound and The Cancer Genome Atlas evaluated its correlation with angiogenesis and prognosis. *In vitro*, cell counting kit-8 (CCK-8), colony formation, transwell, tube formation, and permeability assays examined the effect of HCC cell-derived EVs on human umbilical vein endothelial cells (HUVECs). Subcutaneous tumor and lung metastasis models in nude mice verified it *in vivo* effects. RNA sequencing and databases predicted downstream genes and pathways, and dual luciferase and western blotting assays verified binding and activation. Conditioned medium from treated HUVECs was used on HCC cells, and chemokine levels measured. The CCL20/CCR6 axis effect was studied *in vitro* and *in vivo* by knocking down CCR6.

**Results:**

This study revealed the abnormal upregulation of miR-183-5p in both tissues and plasma EVs from patients with HCC, and its association with unfavorable prognosis. *In vivo* experiments, the promoting effects of miR-183-5p in HCC cell-derived EVs on the progression, metastasis and angiogenesis were verified by employing subcutaneous tumor formation models and lung metastasis models in nude mice. We demonstrated that miR-183-5p in HCC cell-derived EVs induced HUVECs proliferation, migration, angiogenesis and permeability by downregulating SIK1 expression and activating the PI3K/AKT signaling pathway *in vitro*. Moreover, stimulated HUVECs could secrete the chemokine CCL20 and induce HCC progression and metastasis through the CCL20/CCR6 signal pathway *in vitro* and *in vivo.*

**Conclusion:**

The findings indicated that miR-183-5p delivered by EVs from HCC cells is crucial in mediating the communication between HUVECs and HCC cells by modulating the SIK1/PI3K/AKT and CCL20/CCR6 signaling pathways, and EVs-miR-183-5p might be a potential therapeutic target for HCC patients.

## Introduction

As a prevalent malignancy worldwide, primary liver cancer ranks the third (7.8%) leading culprit of cancer-related mortality, with hepatocellular carcinoma (HCC) representing the majority and posing a significant threat to patients’ lives and health (1). Currently, despite the availability of various treatments for patients with HCC, the prognosis remains unfavorable. Therefore, it is crucial to investigate the underlying mechanisms in the pathogenesis of HCC and identify potential target molecules, as this plays a vital role in early diagnosis and prognosis improvement for HCC patients.

Extracellular vesicles (EVs) are membranous vesicles derived from endosomes and cell membranes, capable of transferring RNA, lipids, and proteins for intercellular communication (2). Wang et al. (3) reported that HCC cell-derived S100A10-enriched EVs enhance the stemness and metastasis capacity of HCC cells, up-regulate EGFR, AKT, and ERK signaling, and promote epithelial-mesenchymal transformation (EMT). Fang et al. revealed that miR-1247-3p in HCC cell-derived EVs promotes their conversion to cancer associated fibroblasts (CAFs) by targeting the B4GALT3 gene in fibroblasts. More importantly, activated CAFs also secrete cytokines such as IL-8 and IL-6, which further promote the growth of HCC cells and the ability to resist chemotherapy drugs’ effects (4). Another study has found (5) that miRNA-21 in HCC cell-derived EVs directly targets PTEN, promote the activation of hepatic stellate cells to CAFs, and activate the PDK1/protein kinase B (AKT) signaling pathway. CAFs can further promote the progression of liver cancer by secreting angiogenic cytokines (including VEGF, MMP2, MMP9, bFGF and TGF-β). In addition, HCC cell-derived EVs can polarize macrophages into M2 phenotype. Zhou et al. (6) reported that HCC cell-derived EVs up-regulate TLR4 by inhibiting miR-372-3p via PART1 delivery to promote macrophage M2 polarization in HCC. EVs-mediated transfer of non-coding RNA is involved in various human diseases, particularly cancer, where they exert significant regulatory effects on gene expression involved in tumorigenesis, angiogenesis, metastasis, immune response modulation and drug resistance (2, 7). miRNAs, as a subset of small noncoding RNAs, primarily participate in posttranscriptional regulation (8). Numerous studies have demonstrated that miRNAs, which represent the most highly concentrated cargo within EVs, play a pivotal role in regulating tumor microenvironment (TME) and tumor progression (4, 9–11). For instance, the secretion by HCC cells of EVs containing miR-1247-3p activates CAFs, thereby promoting the lung metastasis of HCC (4). Furthermore, it has been reported that EVs carrying miR-23a-3p derived from M2 macrophages enhance vascular permeability to facilitate HCC metastasis (12). Previous studies have highlighted the pivotal role of EVs-miRNAs in mediating intercellular communication, and therefore, it is crucial to investigate the mechanism by which HCC cell-derived EVs regulate tumor progression.

In this study, we will conduct a systematic study on tissue and plasma samples from HCC patients to explore miRNAs that may be closely related to HCC progression and metastasis. Subsequently, we will further analyze the interaction of miRNAs in HCC cell-derived EVs with human umbilical vein endothelial cells (HUVECs), and elucidate its mechanism of action. The role of HCC cell-derived EVs on HUVECs production and the intrinsic link between this role and HCC progression and metastasis was examined by building an *in vivo* experimental model. This study is expected to help identify biomarkers that can be used to accurately monitor HCC progression and prognosis.

## Materials and methods

### Tissue samples

The study strictly adhered to the principles of the Declaration of Helsinki, and all patients participating in the study were given written informed consent prior to the study to ensure their full understanding and voluntary participation. The human sample study involved in this study has been approved by the Ethics Committee of the Second Affiliated Hospital of Harbin Medical University (Research Ethics Approval Code: KY2020-274). To explore the expression differences of target miRNAs in HCC tissues and adjacent control tissues, we initially collected samples of HCC tissues and distal adjacent control liver tissues (located in different liver lobes from the tumor) from preoperative routine ultrasound-guided tissue biopsies of 20 HCC patients (aged range from 34-73, median 53). Tissue biopsy was performed using 16-G Tru-Cut biopsy needles (Bard, Covington, GA, USA). Additionally, 20 postoperative HCC samples were collected from 20 HCC patients (age range from 38-73, mean 56.7) who underwent contrast-enhanced ultrasound and eventually surgery for verification of the relationship between pathological grading and miR-183-5p expression. None of these patients had received radiotherapy, chemotherapy, or other treatments before these procedures. Prior to all operations, formal informed consent was obtained from each patient or their family members. All the ultrasound-guided biopsy and postoperative pathological tissue samples obtained were divided into two parts: one part was rapidly frozen in liquid nitrogen and stored at -80°C, and the other part was sent to the pathology department for examination by two doctors with over ten years of experience. In case of disagreement, the two doctors would reach a consensus through discussion.

### Plasma sample

10-15ml of whole blood samples were collected from 20 HCC patient’s (age range from 35-72, median 45) elbow vein and placed into an EDTA-containing anticoagulant tube before the patients underwent a fasting contrast-enhanced ultrasound (CEUS) in the morning. Subsequently, Then, the whole blood was centrifuged at 2500 × g with an external pendulum rotor as soon as possible for 15min, the supernatant was carefully absorbed to avoid absorbing the middle layer, and then centrifuged at 2500 × g for 20min. Carefully absorb supernatant (about 5mL plasma) to avoid touching the bottom and side precipitation. The plasma is then stored in an environment with a temperature of -80°C. The same method was used to obtain plasma from 20 healthy controls (age range from 23-67, mean 44.8), and informed consent from each participant was obtained before blood samples were collected.

### CEUS, ultrasound-guided percutaneous biopsies and superb microvascular imaging

The Aplio i900 Color Doppler Ultrasound Diagnostic Device (Canon Medical Systems, Tokyo, Japan) equipped with i8CX1 convex transducer (frequency range 1–8 MHz) and i18LX5 line array transducer (frequency range 8–15 MHz) was utilized to perform CEUS, conventional ultrasound (CUS), SMI, and ultrasound-guided percutaneous biopsies. Initially, the patient assumed a supine or lateral position, whereas the probe was positioned strategically to minimize the interference of respiratory motion and improve the visualization of lesions. The lesions were thoroughly scanned using B-mode ultrasound, and images were saved. Subsequently, the imaging mode was switched to the contrast-enhanced dual-screen mode with a mechanical index set below 0.1. SonoVue (Bracco, Milan, Italy) was rapidly injected into the median cubital vein at a volume of 2.5 mL, followed by 5 mL of normal saline solution while activating timing and dynamic image storage on the ultrasound system. Serial scans of the lesion and surrounding tissue were performed every 15 s for 4–6 min to obtain dynamic CEUS images, which were then saved for further analysis. The quantitative analysis software SonoLiver (TomTec Imaging Systems, Unterschleissheim, Germany) was employed to analyze contrast fragments by placing regions of interest within both the lesion and adjacent liver tissue to generate time-intensity (TIC), time to peak (TTP), and intensity-maximum (IMAX). Following CEUS examination under real-time ultrasound guidance, two physicians collaborated to avoid necrotic areas and blood vessels during biopsy procedures, ensuring accurate sampling from tumor tissue and surrounding normal tissue using biopsy needles. The tissue samples collected were then sent to the pathology department for further analysis. SMI technology can obtain the information of fine blood flow inside the tumor more conveniently, quickly and sensitively without the use of contrast agent. Therefore, we chose to use SMI technology to evaluate the blood flow situation in the subcutaneous tumor of mice. After anesthesia, the subcutaneous tumor was examined from multiple angles using i18LX5 probe, and then the SMI mode was activated. After the image quality is stabilized, the sections with the most abundant blood flow display are stored for later evaluation.

### Extracellular vesicles extraction

EVs separation from blood plasma was performed via size exclusion chromatography (SEC). Sepharose CL-2B columns (Sigma–Aldrich) were assembled using 10 mL syringe with 20μm pore size nylon net (Sigma–Aldrich) at the bottom. Plasma was loaded on top, and 1 mL fractions were eluted. 3 mL of EV-enriched eluates (fractions 4–6) was collected and was centrifuged at 100,000 × g at 4°C for 90 min. Pellets were used for subsequent miRNA extraction.

The collected cell culture medium underwent successive differential centrifugation steps at 300 × g, 4°C for 10 min, 2000 × g, 4°C for 20 min, and 13,000 × g, 4°C for 30 min. After filtering with a 0.22μm syringe filter (Millipore), the supernatant was centrifuged at 100,000 × g, 4°C for 120 min a Beckman Coulter Optima XPN-100 Ultracentrifuge equipped with an SW41Ti rotor. The pellets obtained from the previous step were subjected to a wash with PBS and centrifuged at 100,000 × g, 4°C for 90min. The isolated EVs pellet was subsequently resuspended in PBS. Any EVs not immediately used are stored at −80°C.

### RNA extraction and quantitative real-time polymerase chain reaction

Trizol reagent (#15596026, Invitrogen, USA) was utilized to isolate total RNA from the cell samples. The Molpure Cell/Tissue miRNA Kit (#RC201, Vazyme, China) was utilized to extract total miRNA from EVs, cells and tissue samples. The Transcriptor First Strand cDNA Synthesis Kit (#04897030001, Roche, Germany) was used to reverse the transcription of both miRNA and total mRNA into cDNA. The FastStart Universal SYBR Green Master (ROX) (#04913914001, Roche, Germany) was utilized for qRT-PCR analysis to determine RNA expression levels. The results were normalized using GAPDH, small RNA U6, or Cel-miR-39-3p expression levels. The complete primer sequence for this study is provided in **Supplementary Table S1**.

### EVs characterization, identification and uptake assay

The size and shape of EVs were characterized using transmission electron microscopy (TEM) and nanoparticle tracking analysis (NTA). 1μL EVs solution extracted from each group was absorbed, 9μL PBS buffer was added, and thoroughly mixed. The mixture was evenly and appropriately added to the copper mesh with 200 mesh liquid carrier, and then the copper mesh was placed at room temperature for 1min so that the sample could be naturally adsorbed to the surface of the mesh. Then use filter paper to remove excess liquid from the copper mesh. Further, 10μL of 2% uranoxy acetate reagent was added to the copper mesh and left for 1 minute to make the dye fully permeate and combine with EVs. The excess dye was carefully absorbed with filter paper again and washed with PBS 3 times to avoid affecting the final image quality. The dried copper mesh was carefully placed in the transmission electron microscope for observation and image recording.

EVs samples were obtained from each group, diluted with DPBS without nanoparticles to a concentration of 10^6^-10^7^ particles/mL, and thoroughly mixed, so that EVs presented a single distribution. The sample is evenly dripped into the detection hole of the sample chamber to avoid bubbles. The data is collected, analyzed and recorded using Zeta View PMX 110 (Particle Metrix, Meerbusch, Germany) (Zeta View 8.04.02 SP2).

To identify EVs, western blotting assay was employed to analyze the expression levels of CD9, CD81, and Alix, while Calnexin and GM130 served as a negative control for EVs. After the identification, the HCC cell-derived EVs were mixed and marked with a working solution of PKH26 (Sigma-Aldrich, USA), incubated for 5 min without light, and then centrifuged at 100,000 × g for 90 min twice. Subsequently, the PKH26-labeled HCC cell-derived EVs were co-cultured with HUVECs for 6h and visualized under a fluorescence microscope (Nikon, Tokyo, Japan).

### Cell lines and culture conditions

Four HCC cell lines (MHCC97H, Huh7, Huh1, and Hep3B), one normal liver cell line (THLE-2), and HUVEC were purchased from Procell Life Science and Technology (Wuhan, China). According to the specific culture requirements of different cell lines, a culture medium (CM) containing 10% fetal bovine serum (FBS) corresponding to the cell line was used for cell culture. The method of obtaining CM was as follows: centrifuge the HUVEC supernatant at 2000rpm for 10min, then filter through a 0.22μm filter, and the ratio of CM to fresh medium was 1:1. The cells used to extract supernatant EVs were cultured in serum without EVs. EVs-free FBS was obtained by centrifuging FBS for 18h at 4°C, 100,000 × g (a Beckman Coulter Optima XPN-100 Ultracentrifuge equipped with an SW45Ti rotor).

### Cell transduction

Lentiviral transduction techniques from Genepharma (Shanghai, China) were employed to establish HUVEC lines with stable overexpression and down-regulation of SIK1 and C-C chemokine receptor 6 (CCR6). Based on the experimental requirements, miR-183-5p mimics/NC or inhibitors/NC (Genepharma, Shanghai, China) were transfected into the Hep3B, Huh7, and HUVEC lines to obtain overexpressing or knockdown miR-183-5p cell lines and their negative controls. All involved sequences are shown in **Supplementary Tables S2**–**S5**.

### Cell proliferation assays

The experimental cells and the negative control group were inoculated at an equal density into a 96-well plate. Cell Counting Kit-8 (CCK-8; #C0038, Beyotime, China) solution on culture days 1, 2, 3, 4, and 5 was employed to evaluate the cell proliferation capacity in the respective wells. The plate colony formation assay was utilized to assess the proliferative ability of the cells. The cells were placed in a 6-well plate and incubated for two weeks before fixation. Subsequently, crystal violet staining solution (#C0121, Beyotime, China) was applied to stain each well. Furthermore, images were captured, and a quantitative analysis was performed.

### Cell migration and invasion assays

In migration experiments, 3–8 × 10^4^ Huh7, Hep3B, or HUVECs were seeded onto the upper layer of transwell chambers with their respective serum-free medium, while the lower chamber was filled with corresponding medium supplemented with 10% serum. Unlike the migration assay, the upper chamber should be coated with a 1:8 diluted matrix gel (#356234, Corning Corporation, USA) before the invasion assay. After incubation for 24–48 h, the cells on the transwell chamber were fixed with 4% paraformaldehyde solution, the unpenetrated cells were wiped off with a cotton swab, and then the penetrated cells were stained with crystal purple staining solution (#C0078, Beyotime, China). The cells that pass through are photographed and counted. The wound healing assay was also used to test the migration ability of the cells, allowing the corresponding cells to be overgrown in the 6-well plate. The fully confluent cells were scratched with the tip of a 200μL pipette gun, and then the cells were replaced with serum-free medium for 24 h. The degree of scratch healing was observed with an inverted microscope, and photos were recorded at 0 and 24 h.

### Endothelial cell permeability assay

The endothelial cells were evenly spread in transwell chambers (8μm), and CM (containing different treated EVs) were added to both upper and lower chambers. After the endothelial cells were fully spread in the upper chamber, living cell tracer (#40721ES50, Yeasen, Shanghai, China) labeled Huh7/Hep3B cell suspension (serum-free medium) was added to the upper chamber, and the lower chamber was replaced with a medium containing 10% FBS (without EVs). After continuous culture for 12–24 h, the cells were cultured. The upper compartment cells were erased and Huh7/Hep3B cells across the transwell compartment were photographed using fluorescence microscopy.

### Tube formation assay

A total of 150μL Matrigel (#356234, Corning, USA) was added to each well of the 48-well plate, ensuring no bubbles. The plate was then placed in a 37°C incubator. After 1 h, HUVEC suspensions subjected to different treatments were added to 48-well plates with 7 × 10^4^ cells/wells. Cells were incubated at 37°C for 2–5 h, then stained with Calcein-AM (#CA1630, Solarbio, Shanghai, China) and photographed.

### Western blotting assay and antibodies

Total cell protein was lysed using radio-immunoprecipitation assay lysis buffer (#P0013B, Beyotime, China). An enhanced bicinchoninic acid protein assay kit (#P0010, Beyotime, China) was used to quantify the protein of the supernatant obtained by lysis. For Western blotting analysis, sufficiently denatured protein samples were isolated on sodium dodecyl sulfate-polyacrylamide gel electrophoresis with a concentration of 10% or 12.5% (#PG112, PG113, Epizyme, China) and subsequently transferred to a polyvinylidene fluoride membrane (#IPFL00010, Merkmillibeau, Germany). Furthermore, 5% skim milk powder was blocked in the membrane at 37°C for 2 h and incubated with the corresponding primary antibody at 4°C overnight. After the membrane was cleaned, the second antibody of the corresponding species was incubated at room temperature for 2 h, and the protein signal was detected using the enhanced chemiluminescence kit (#MA0186, Meilunbio, China). The Bio-Rad ChemiDoc MP system was used to acquire and analyze the images. **Supplementary Table S6** shows the details of the antibodies used.

### Luciferase reporter assay

Dual luciferase reporter gene assay was used to verify the direct interaction between SIK1 and miR-183-5p. The 3’-UTR sequence of SIK1 (containing the binding prediction site of miR-183-5p) and the specific mutant (MUT) sequence were cloned into the dual luciferase reporter gene vectors and named as pmirRB-SIK1-WT and pmirRB-SIK1-MUT reporter gene vector (Riobio, Guangzhou, China), respectively (1). miR-183-5p mimics/NC or miR-183-5p inhibitor/NC plasmid (Genepharma, Shanghai, China) was co-transfected into HUVEC with pmirRB-SIK1-WT/pmirRB-SIK1-MUT; (2) pmirRB-SIK1-WT/pmirRB-SIK1-MUT plasmid was transfected with HUVEC treated with miR-183-5pinhibitor Huh7/EVs or miR-183-5p mimics Hep3B/EVs (Genepharma, Shanghai, China). 48 h after transfection, the change of luciferase activity was detected using a dual luciferase assay kit (#E1910, Promega, USA) according to the instructions.

### Enzyme-linked immunosorbent assay

The Bio-Plex Pro 40-Plex Human Chemokine Panel (#171AK99MR2, Bio-Rad, CA, USA) was utilized to screen chemokines associated with angiogenesis. To evaluate the effect of HCC cell-derived EVs on chemokine (C–C motif) ligand 20 (CCL20) expression in HUVECs, ELISA was performed using a Human CCL20 ELISA Kit (#CB12912-Hu, Coibo Biotechnology Co., China) based on the instructions.

### Animals

The animal experiments were designed and carried out in strict accordance with the National Institutes of Health (NIH) Guidelines for the Care and Use of Laboratory Animals. The animal experiments were approved by the Animal Ethics Committee of the Second Affiliated Hospital of Harbin Medical University (Research Ethics Approval Code: SYDW2023-025). Then, the experiments were conducted following the approved protocol and relevant ethical and scientific standards. The animals in this study were randomly allocated to different experimental groups, and the investigators were not blinded to the group allocations. Sample size justification for animal studies was based on preliminary experiments. The manuscript was prepared following the ARRIVE guidelines for animal research.

In order to explore the role of miR-183-5p in HCC cell-derived EVs, we used Hep3B cells to construct mice subcutaneous tumor formation model and lung metastasis model for verification. After Hep3B cells were made into a suspension, inject into the right dorsal subcutaneous region or the tail vein of 4-week-old male nude mice (BALB/c) that are specific pathogen free (SPF). 5 mice were included in each group, and the injection dose was set to 5 × 10^6^ cells/mice. Then, the mice were treated with specific Hep3B-derived EVs via tail vein injection every 4 days (Hep3B miR-183-5p mimics-EVs or Hep3B miR-183-5p NC-EVs). The subcutaneous tumor volume of the mice was measured every 5 days, and the mice were sacrificed at the end of 4 weeks. The tumor volume was calculated by applying the formula (L × W²)/2. In this formula, L represents the maximum diameter of the tumor, and W represents for the diameter perpendicular to the longest one. The tumor tissues were weighed and immunohistochemistry (IHC) staining were performed. After 8 weeks, the lung tissues of mice in the lung metastasis model group were taken out for hematoxylin-eosin (H&E) staining, and the number of lung metastasis nodules was recorded.

*In vivo* validation of the role of HCC cell-derived EVs-activated HUVECs in promoting HCC progression, Hep3B cells were co-cultured with CM from HUVECs pretreated with miR-183a-5p mimics-loaded or control EVs for 48 h. Hep3B cells were then injected into the subcutaneous tissue of the mice, and the subcutaneous tumor volume of the mice was recorded every 5 days. The mice were euthanized 4 weeks later, and the tumor tissue was retained for weighing and IHC staining.

To verify the role of CCL20/CCR6 axis in promoting HCC progression and metastasis, the following groups of cell suspensions were injected into the subcutaneous tissue or tail vein of mice, respectively, to establish mice subcutaneous tumor formation model and lung metastasis model: (1) untreated Hep3B cells; (2) Hep3B cells cultured with HUVEC miR-183-5p mimics/CM + IgG for 48h; (3) Hep3B cells cultured with HUVEC miR-183-5p mimics/CM + CCL20 antibody (Ab) for 48h; (4) sh-NC Hep3B cells cultured with HUVEC miR-183-5p mimics/CM for 48h; (5) sh-CCR-6 Hep3B cells cultured with HUVEC miR-183-5p mimics/CM for 48h. The subcutaneous tumor volume was recorded every 5 days from the injection date. After 4 weeks, the tumors of the subcutaneous tumor model group of mice were examined using SMI technology, then the mice were euthanized, and the tumor tissues were kept for weighing and IHC staining. After 8 weeks, the lung tissues of mice in the lung metastasis model group were taken out for H&E staining, and the number of lung metastasis nodules was recorded.

### IHC staining

The samples were dewaxed and hydrated with gradient alcohol. Then antigen repair device (microwave oven) combined with sodium citrate antigen repair solution (#P0081, Beyotime, China) was used to improve antibody recognition of target antigen. To mitigate the effects of nonspecific antigens, endogenous peroxidase blocker H2O2 (#PV6000, ZSGB-BIO, China) was introduced. Subsequently, tissue samples were sealed with 5% bovine serum albumin (#ST023, Beyotime, China). The samples were incubated with the primary antibody at 4°C overnight to ensure a firm bond between the antibody and the antigen. After that, it was incubated with suitable secondary antibodies (#PV6000, ZSGB-BIO, China) and color development was carried out. The addition of DAB (#P0202, Beyotime, China) triggers a brown reaction, and hematoxylin (#C0107, Beyotime, China) re-stains the nucleus, providing a clear structural contrast. The staining intensity was divided into negative (0), weak (1), medium (2) and strong (3). Meanwhile, the percentage of positive cells was 0-25% (1), 26-50% (2), 51-75% (3) and 76-100% (4), respectively. Finally, the IHC score of each tissue was calculated by multiplying the staining intensity score with the percentage of positive cells. **Supplementary Table S6** shows the details of the primary antibodies used.

### H&E staining

After paraffin removal and moisture restoration, the H&E staining kit (#C0105S, Beyotime, China) was used to stain tissue samples based on instructions, and then sections were sealed with neutral adhesive, followed by microscopic observation and image retention.

### RNA sequencing

RNA sequencing was performed and the results were analyzed by Shanghai OE Biotech Co.,Ltd (Shanghai, China). Briefly, total RNA was extracted from HUVECs using the TRIzol reagent (#15596206, Invitrogen, CA, USA). RNA integrity was assessed using the Agilent 2100 Bioanalyzer (Agilent Technologies, Santa Clara, CA, USA). Then the libraries were constructed using VAHTS Universal V6 RNA-seq Library Prep Kit according to the manufacturer’s instructions. The libraries were sequenced on a llumina Novaseq 6000 platform and 150 bp paired-end reads were generated. The initial data were purified to obtain high-quality, clean data. The raw data were deposited in the Gene Expression Omnibus database under accession number GSE289187.

### Statistical analysis and reproducibility

Statistical analysis was conducted using GraphPad Prism software (version 9.0; La Jolla, CA, USA). The data were presented as means ± standard deviation (SD) or depicted as box plots indicating the maximum, median, and minimum values. For the comparison between two groups, we employed Student’s t-test (two-tailed) or Mann-Whitney U test. To visualize statistical differences in survival, the Kaplan-Meier curve was utilized and Log-Rank (Mantel-Cox) test was performed. Microscopy images shown are representative from three independent experiments. All cell experiments were performed in triplicate, with each experiment repeated at least three times. (*P < 0.05, **P < 0.01, ***P < 0.001).

## Results

### Abnormal expression of miR-183-5p is related to HCC progression and poor prognosis

**Figure 1:**
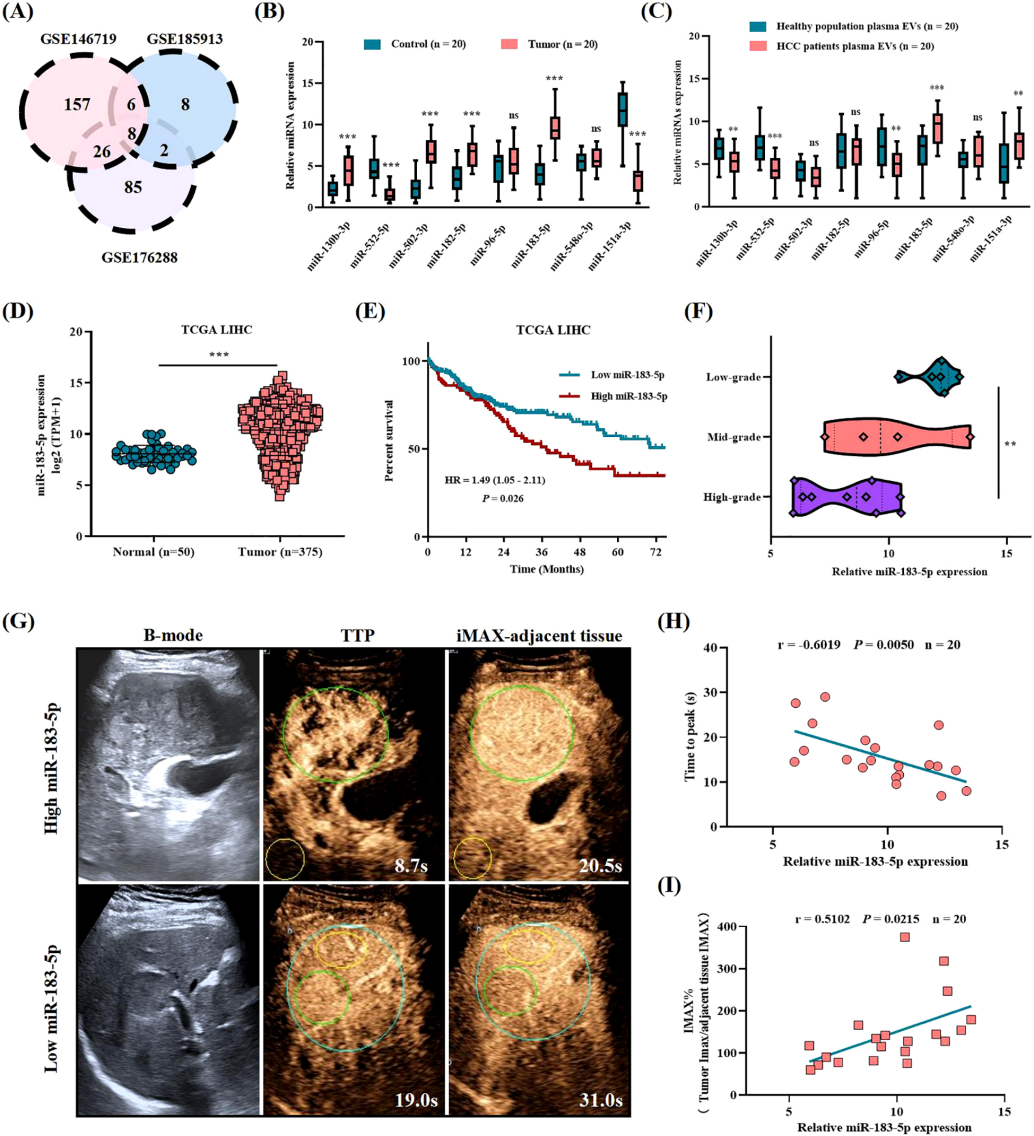
The abnormal expression of miR-183-5p is related to HCC progression and poor prognosis. **(A)** Differentially expressed miRNAs in HCC tissues compared with normal tissues were identified in three GEO databases (GSE146719, GSE185913, and GSE176288). Distinct color regions represent different datasets, while overlapping regions indicate miRNA intersections. **(B)** The expression levels of eight miRNAs were assessed in HCC tissues (n = 20) and adjacent control liver tissues (n = 20). Data are expressed as box plots indicating the maximum, median, and minimum values. **(C)** The expression levels of eight miRNAs were assessed in plasma EVs from patients with HCC (n = 20) and healthy population plasma EVs (n = 20). Data are expressed as box plots indicating the maximum, median, and minimum values. **(D)** The expression of miR-183-5p in HCC tissues (n = 50) was higher than that in normal tissues (n = 375) in the TCGA database. Data are presented as means ± SD. **(E)** Higher levels of miR-183-5p were associated with a significantly worse overall survival outcome in the TCGA database. **(F)** Based on the patients with HCC in our center, the high expression of miR-183-5p predicted HCC pathological stage deterioration (n = 20). Data are expressed as box plots indicating the maximum, median, and minimum values. **(G)** B-mode ultrasound and CEUS images of patients with HCC showing high and low miR-183-5p expression in plasma EVs. The green circle is the tumor area, and the yellow circle is the adjacent liver tissue of the control. **(H)** The TIC analysis showed that the miR-183-5p expression of plasma EVs was negatively correlated with the TTP parameters (n = 20). **(I)** The TIC analysis showed that the miR-183-5p expression of plasma EVs was positively correlated with the IMAX parameters (n = 20). This figure shows the statistical data of three independent experiment. **P < 0.01, ***P < 0.001. ns, not significant.

To begin with, we searched three Gene Expression Omnibus (GEO) databases (GSE146719, GSE185913, and GSE176288) and found that eight miRNAs (including miR-130b-3p, miR-532-5p, miR-502-3p, miR-182-5p, miR-96-5p, miR-183-5p, miR-548-3p, and miR-151a-3p) expressed differently in HCC tissues and control tissues (**Figure 1A**). We also verified the expression levels of these eight miRNAs in HCC tissues and adjacent control liver tissues by performing qRT-PCR on 20 pairs of tissues in our center and revealed that six miRNAs (including miR-130b-3p, miR-532-5p, miR-502-3p, miR-182-5p, miR-183-5p, and miR-151a-3p) showed significant differences in the tissues qRT-PCR verification (**Figure 1B**). Recent studies showed that EVs play an important role in the intercellular communication of HCC. Therefore, we also performed qRT-PCR verification on miRNA expression levels isolated from plasma EVs obtained from patients with HCC and healthy population. The results showed that five miRNAs were differentially expressed in plasma EVs of HCC patients and healthy population, namely miR-130b-3p, miR-532-5p, miR-96-5p, miR-151a-3p, and miR-183-5p, respectively. And miR-183-5p was the only miRNA highly expressed in both tissues and plasma EVs of HCC patients, suggesting it might contribute to HCC progression as a crucial role (**Figure 1C**). The cancer genome atlas (TCGA) demonstrated that miR-183-5p expression is significantly upregulated in HCC tissues compared to control tissues (**Figure 1D**) and elevated miR-183-5p expression was related to poor prognosis in HCC patients (**Figure 1E**). Our independent validation using 20 HCC tissues further confirmed that high miR-183-5p expression was associated with a lower pathological grade (**Figure 1F**). The TIC analysis of these 20 patients with HCC revealed a negative correlation between the miR-183-5p expression of plasma EVs and TTP parameters, which were negatively associated with vascular density (13, 14) (**Figures 1G, H**). Additionally, a positive association was observed between the miR-183-5p expression of plasma EVs and IMAX parameters, which were positively correlated with microvascular density (13, 15) (**Figures 1G, I**). The results suggested upregulation of miR-183-5p is related to HCC progression, and therefore, the function and molecular mechanism for miR-183-5p in HCC deserves further exploration.

### High expression of miR-183-5p in HCC cell-derived EVs promotes angiogenesis

**Figure 2:**
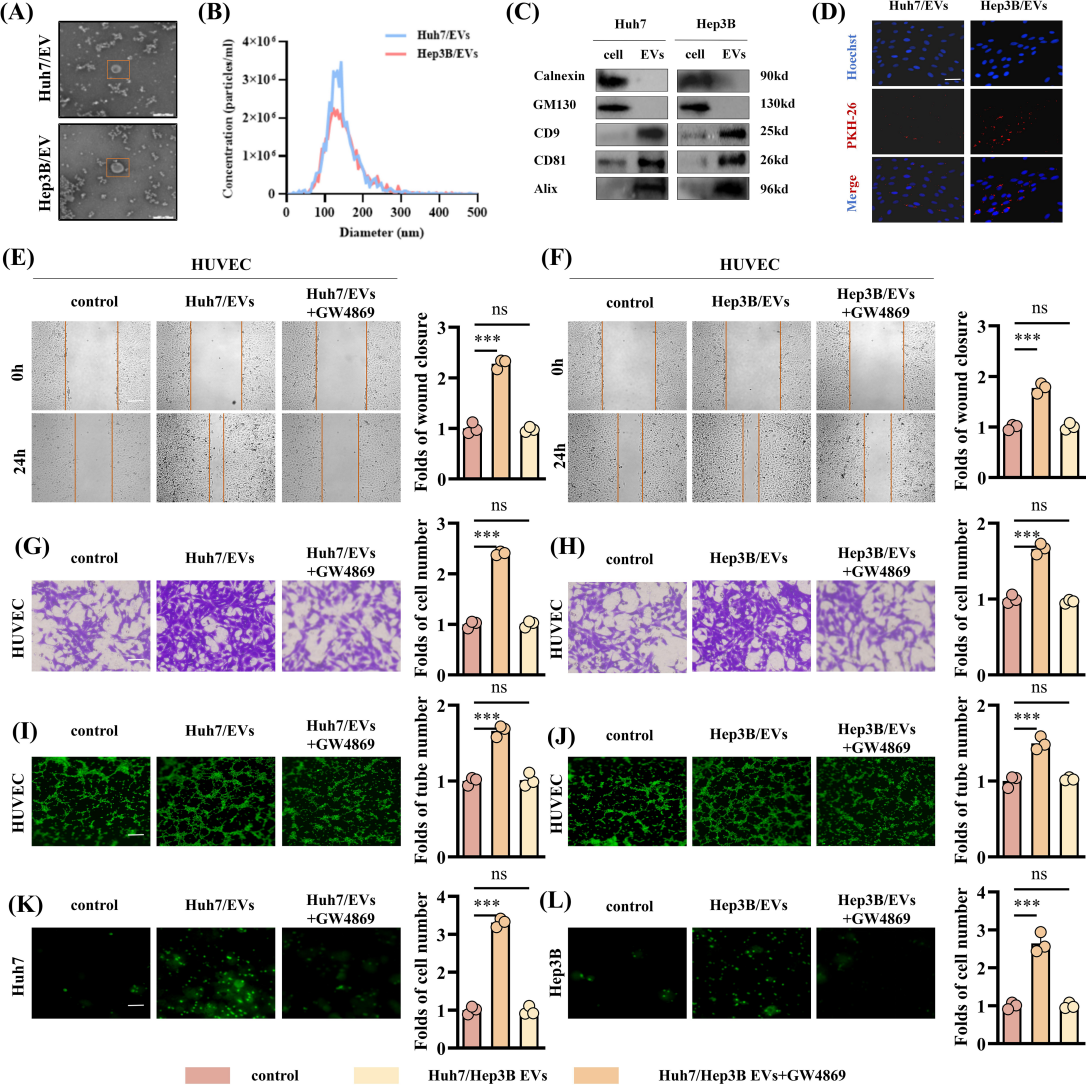
HCC cell-derived EVs can promote angiogenesis of HUVECs. **(A)** The TEM images of EVs from Huh7 and Hep3B cell lines. Scale bar: 200μm. **(B)** The NTA showed the particle size of EVs derived from Huh7 and Hep3B cell lines. **(C)** Western blotting assay was performed to detect the expression of the negative EVs biomarker (Calnexin and GM130) and typical EVs biomarkers (CD9, CD81 and Alix) in EVs derived from the Huh7 and Hep3B cell lines. **(D)** Uptake of HUVECs after incubation with PKH-labeled EVs. Scale bar: 100μm. **(E-H)** The EVs derived from HCC cell lines show an enhanced ability to promote HUVECs migration. Data are presented as means ± SD (n = 3). **(E, F)** Scale bar: 100μm. **(G, H)** Scale bar: 25μm. **(I, J)** The EVs derived from HCC cell lines show an enhanced ability to promote HUVECs angiogenesis. Data are presented as means ± SD (n = 3). Scale bar: 50μm. **(K, L)** The EVs derived from HCC cell lines show an enhanced ability to promote HUVEC permeability. Data are presented as means ± SD (n = 3). Scale bar: 25μm. Statistical data presents in this figure show means ± SD of three times of independent experiments. ***P < 0.001. ns, not significant.

Next, we evaluated the miR-183-5p expression level in EVs released by four HCC cell lines and one normal liver cell line and the results revealed that the miR-183-5p expression level was significantly higher in EVs derived from HCC cells than normal cells (**Supplementary Figure S1**). EVs originating from tumor cells play a crucial role in driving cancer progression and metastasis by facilitating the transfer of bioactive molecules among diverse cell populations within the TME. We first asked whether HCC cell-derived EVs have an effect on HUVECs. To this end, we selected Huh7 and Hep3B cell lines to isolate and characterize their respective supernatant EVs for further analysis. The EVs, obtained from Huh7 and Hep3B cells, were identified using TEM and confirmed to be less than 200nm in diameter (**Figures 2A, B**). In addition, the western blotting assay confirmed positive expressions of CD8, CD81, and Alix as EVs biomarkers, while no detectable expression of Calnexin and GM130 as negative marker was observed (**Figure 2C**). As shown in **Figure 2D**, when cocultured with HUVECs for 6h, HCC cell-derived EVs labeled with PKH were observed within the HUVECs. In addition, compared with the control group, HCC cell-derived EVs promoted HUVECs proliferation (**Supplementary Figures S2A, B**), migration (**Figures 2E–H**), angiogenesis (**Figures 2I, J**) and permeability (**Figures 2K, L**), while the EVs derived from the same amount of HCC cells treated with GW4869 significantly reduced the stimulation effect of HUVECs.

**Figure 3:**
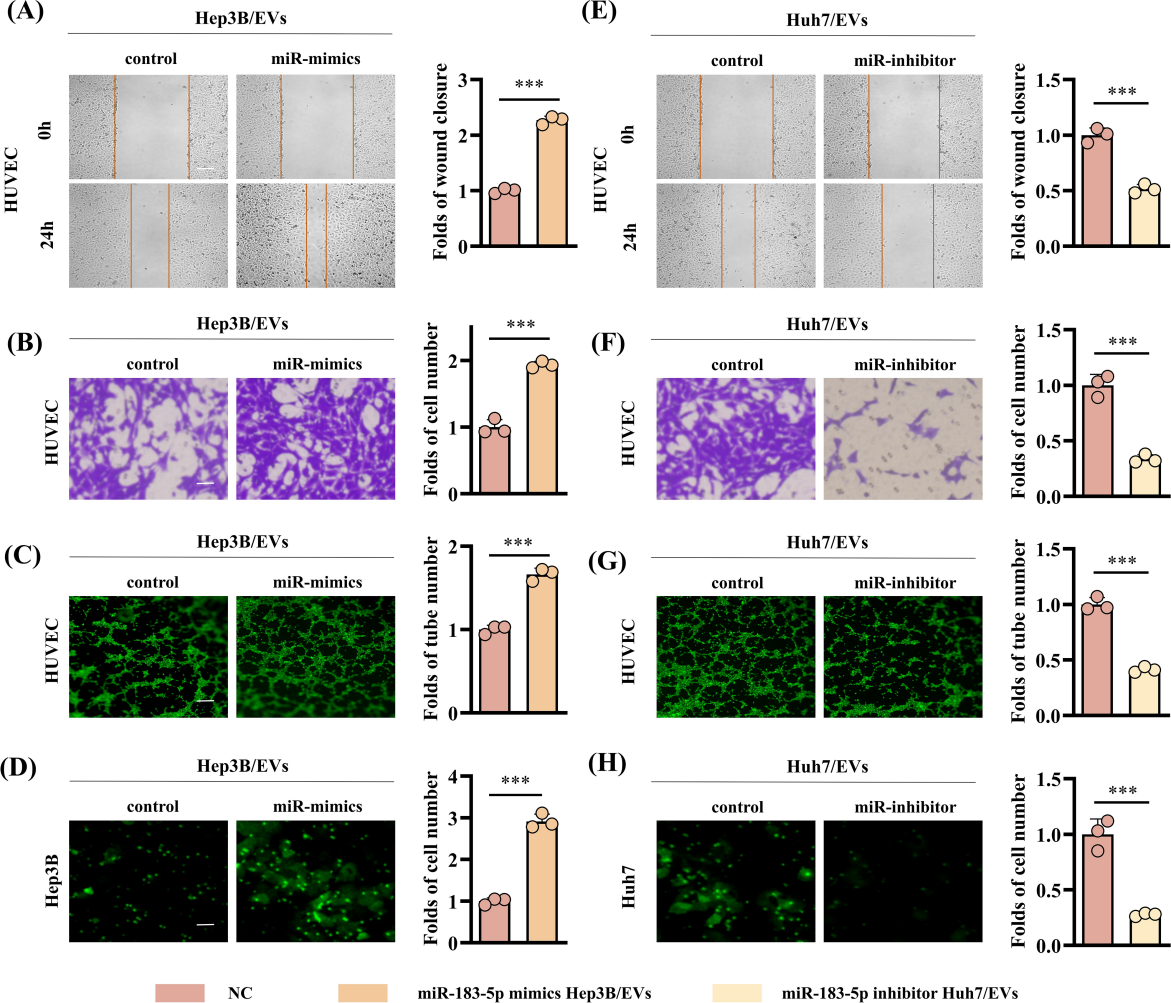
High expression of miR-183-5p in HCC cell-derived EVs promotes angiogenesis. **(A, B)** The miR-mimics Hep3B/EVs significantly promote migration capacity of HUVECs. Data are presented as means ± SD (n = 3). **(A)** Scale bar: 100μm. **(B)** Scale bar: 25μm. **(C)** The miR-mimics Hep3B/EVs significantly promote angiogenesis capacity of HUVECs. Data are presented as means ± SD (n = 3). Scale bar: 50μm. **(D)** The miR-mimics Hep3B/EVs significantly promote permeability capacity of HUVECs. Data are presented as means ± SD (n = 3). Scale bar: 25μm. **(E, F)** The miR-inhibitor Huh7/EVs significantly inhibit migration capacity of HUVECs. Data are presented as means ± SD (n = 3). **(G)** The miR-mimics Huh7/EVs significantly inhibit angiogenesis capacity of HUVECs. Data are presented as means ± SD (n = 3). **(H)** The miR-mimics Huh7/EVs significantly inhibit permeability capacity of HUVECs. Data are presented as means ± SD (n = 3). Statistical data presents in this figure show means ± SD of three times of independent experiments. ***P < 0.001.

In order to further explore whether the EVs-induced angiogenesis of HCC cells is affected by the high expression of miR-183-5p, we transfected miR-183-5p mimics and miR-183-5p inhibitor into Hep3B and Huh7 cells, respectively, and extracted EVs from the cell supernatant. The transfection efficiency was confirmed by qRT-PCR (**Supplementary Figures S3A, B**). Compared with the control group, the miR-mimics Hep3B/EVs significantly promoted proliferation, migration, angiogenesis and permeability capacity of HUVECs (**Figures 3A–D** and **Supplementary Figures S4A, B**), whereas miR-inhibitor Huh7/EVs markedly reduced these abilities (**Figures 3E–H**). The results above suggested that miR-183-5p in HCC cell-derived EVs could promote cell proliferation, migration, angiogenesis and permeability of HUVECs.

### miR-183-5p in HCC cell-derived EVs promoted HCC progression *in vivo*

**Figure 4:**
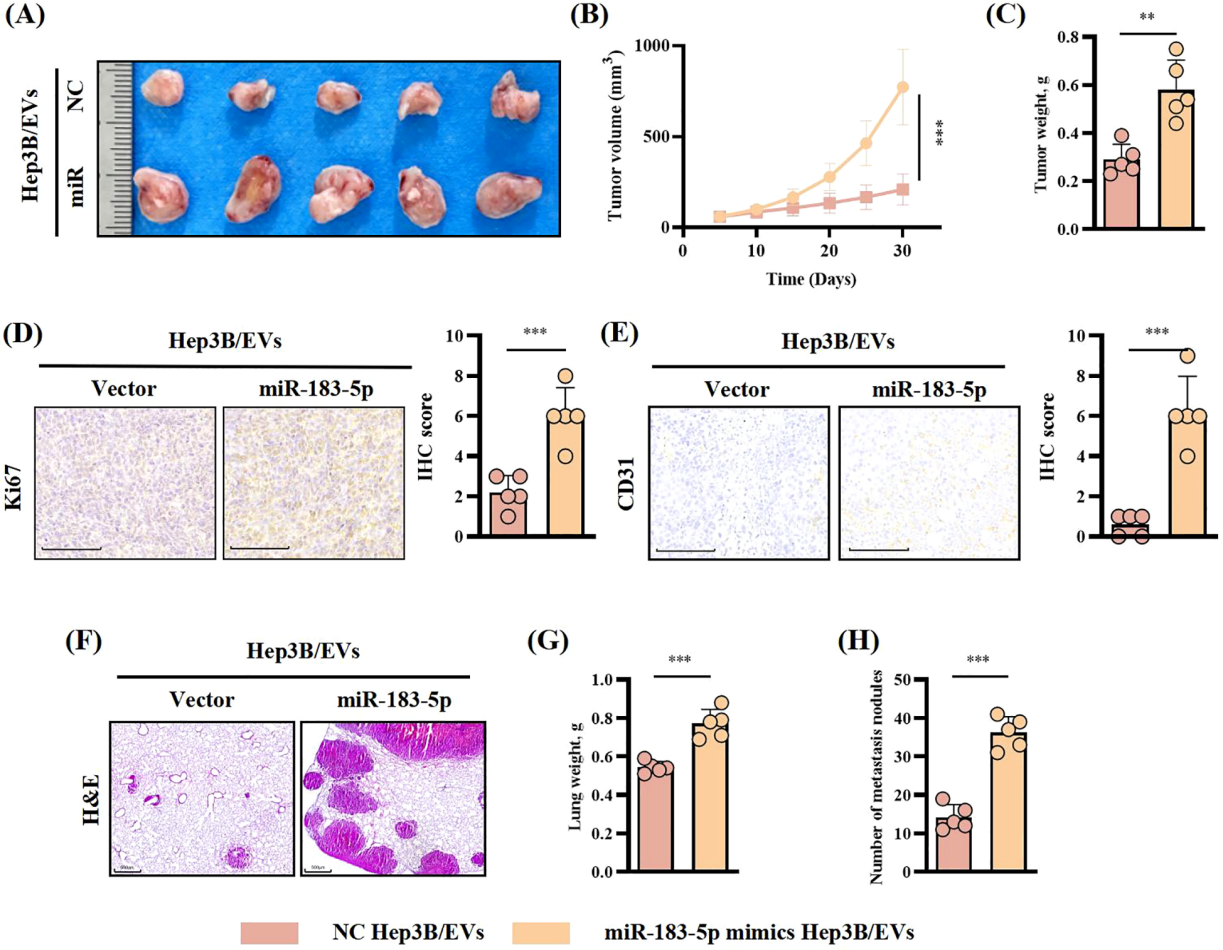
miR-183-5p in HCC cell-derived EVs promoted HCC progression *in vivo.* **(A, B)** The tumor volumes were determined in the xenograft model for both the group with tail vein injection of NC-Hep3B/EVs and that with tail vein injection of miR-183-5p mimics-Hep3B/EVs. Data are presented as means ± SD (n = 5 in each group). **(C)** The tumor weights were determined in the xenograft model for both the group with tail vein injection of NC-Hep3B/EVs and that with tail vein injection of miR-183-5p mimics-Hep3B/EVs. Data are presented as means ± SD (n = 5 in each group). **(D, E)** Relative expression level and representative IHC staining images of Ki67 and CD31 for indicated tumor tissues. Data are presented as means ± SD (n = 5 in each group). Scale bar: 100μm. **(F)** Representative H&E staining of mice lung sections showing tumor lesions. Scale bar: 500μm. **(G, H)** Lung weights and the number of metastatic nodules in the lungs were quantified. Data are presented as means ± SD (n = 5 in each group). **P < 0.01, ***P < 0.001.

In order to explore the role of miR-183-5p in HCC cell-derived EVs *in vivo*, we constructed a mice subcutaneous tumor model and lung metastasis model with Hep3B cells for verification. First, Hep3B cells were injected into mice by subcutaneous injection or through the tail vein, after which the mice were treated with specific Hep3B/EVs tail vein injections every 4 days. Results as shown in **Figures 4A–C**, the injection of miR-183-5p mimics-Hep3B/EVs significantly promoted the growth of subcutaneous tumors in mice, and the volume and weight of tumors significantly increased compared with the control group. IHC staining further showed that the levels of Ki-67 and CD31 expression in tumor tissues increased significantly (**Figures 4D, E**), indicating that miR-183-5p promoted the proliferation and angiogenesis of tumor cells through EVs. In addition, in the lung metastasis model, the number of metastatic nodules in the lungs and the lung weight of mice receiving miR-183-5p mimics/EVs injection significantly increased compared to the control group (**Figures 4F–H**). These results suggest that miR-183-5p in HCC cell-derived EVs accelerates the growth of HCC and significantly promotes HCC metastasis.

### miR-183-5p directly targets SIK1 to activate PI3K/AKT signaling pathway in HUVECs

**Figure 5:**
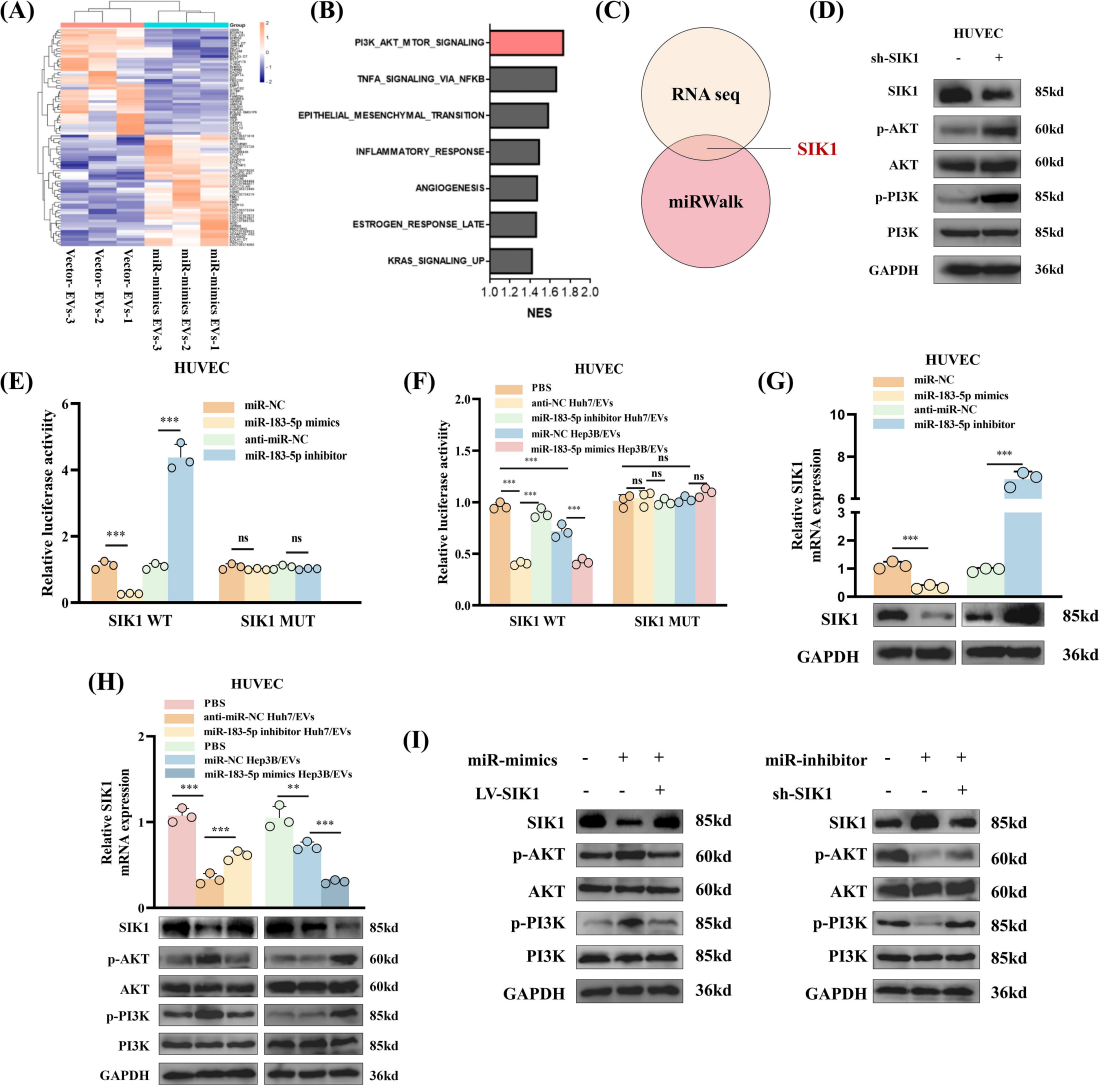
miR-183-5p directly targets SIK1 to activate PI3K/AKT signaling pathway in HUVECs. **(A)** Heat map was shown to identify up- or downregulated transcripts obtained from RNA-seq data for HUVECs after cocultured with Hep3B-vector/EVs or Hep3B-miR-183-5p mimics/EVs. **(B)** Gene set enrichment analysis (GSEA) showed that the PI3K/AKT signaling pathway was notably activated in HUVECs after treated with Hep3B-miR-183-5p mimics/EVs. **(C)** The 3’-UTR of SIK1 contained potential binding sites for miR-183-5p according to the results of RNA seq and miRWalk database. **(D)** Knocking down SIK1 could significantly promote the activation of PI3K/AKT signaling pathway in HUVECs. Three biological replicates. **(E)** A luciferase reporter gene activity assay was performed to determine the effects of miR-183-5p mimics or miR-183-5p inhibitors on the luciferase activity of the 3’-UTR of the WT/MUT SIK1 gene. Data are presented as means ± SD (n = 3). **(F)** A luciferase reporter gene activity assay was performed to determine the effect of miR-183-5p in EVs from Huh7 and Hep3B cell lines transfected with miR-183-5p inhibitor or miR-183-5p mimics on the luciferase activity of the 3’-UTR of the WT/MUT SIK1 gene in HUVECs. Data are presented as means ± SD (n = 3). **(G)** The effects of miR-183-5p mimics and miR-183-5p inhibitors on the expression of SIK1 in HUVECs were evaluated. qRT-PCR data are presented as means ± SD (n = 3). Western blotting assay were performed three biological replicates. **(H)** The effects of miR-183-5p in EVs derived from HCC cells on the expressions of SIK1, p-AKT, AKT, p-PI3K and PI3K in HUVECs were detected using qRT-PCR and western blotting assays. qRT-PCR data are presented as means ± SD (n = 3). Western blotting assay were performed three biological replicates. **(I)** The HUVECs were transfected with LV-SIK1/sh-SIK1 and miR-183-5p mimics/miR-183-5p inhibitors. The expressions of SIK1, p-AKT, AKT, p-PI3K and PI3K in HUVECs were detected using a western blotting assay. Western blotting assay were performed three biological replicates. Statistical data presents in this figure show means ± SD of three times of independent experiments. **P < 0.01, ***P < 0.001. ns, not significant.

The findings from this study suggested that miR-183-5p in HCC cell-derived EVs plays a role in promoting the cell proliferation, migration, angiogenesis and permeability of HUVECs. However, the precise mechanism underlying these effects remains unclear. To explore the target genes of miR-183-5p in HUVECs, we performed an RNA-seq for HUVECs after cocultured with Hep3B-vector/EVs or Hep3B-miR-183-5p mimics/EVs. The results revealed that 65 genes were upregulated while 118 genes were downregulated in HUVECs after cocultured with Hep3B-miR-183-5p mimics/EVs (|log2FC| > 0.5, *P* < 0.05) (**Figure 5A**). After treated with Hep3B-miR-183-5p mimics/EVs, the PI3K/AKT signaling pathway was notably activated in HUVECs (**Figure 5B**). Among the significantly different expressed genes, we found the 3’-UTR of SIK1 contained potential binding sites for miR-183-5p according to the miRWalk database (**Figure 5C**), and it was a pivotal result for us as SIK1 has been documented to inhibit tumor progression in both thyroid and breast cancer (16, 17). A published study has shown that SIK1 inhibits PI3K/AKT pathway activation in HCC cells by promoting reactive oxygen species (ROS) production (18). To verify whether SIK1 has a similar function in HUVECs, we performed ROS generation experiments on HUVECs with SIK1 knockdown and overexpression. As shown in **Supplementary Figures S5A, B**, knocking down SIK1 can inhibit the generation of ROS in HUVECs, while overexpression of SIK1 has the opposite result. Additionally, we investigated whether the expression of SIK1 could influence the activation of the PI3K/AKT pathway. As expected, knocking down SIK1 could significantly promote the activation of PI3K/AKT signaling pathway (**Figure 5D**). Therefore, SIK1 was selected for further investigation in this study. Then, to explore the potential direct interaction between SIK1 and miR-183-5p, we employed a wild-type (WT) double luciferase reporter vector (pmirRB-SIK1-WT) containing the anticipated binding site for miR-183-5p and a MUT double luciferase reporter vector (pmirRB-SIK1-MUT) with a mutation in the miR-183-5p binding site, transfected into HUVECs along with the miR-183-5p mimics or miR-183-5p inhibitor. The results showed that the co-transfection of miR-183-5p mimics and miR-183-5p inhibitor with pmirRB-SIK1-WT significantly modulated the relative luciferase activity compared with the negative control group, while no remarkable changes in luciferase activity were detected when co-transfecting with the MUT vector pmirRB-SIK1-MUT (**Figure 5E**). Similar results in luciferase activity were observed upon transfection of pmirRB-SIK1-WT or pmirRB-SIK1-MUT into HUVECs treated with Huh7-miR-183-5p inhibitor/EVs or Hep3B-miR-183-5p mimics/EVs (**Figure 5F**), thereby confirming that SIK1 in HUVECs is a direct target of miR-183-5p of HCC cell-derived EVs. Moreover, transfection with miR-183-5p mimics or miR-183-5p inhibitors significantly reduced or elevated the mRNA and protein levels of SIK1 in HUVECs, respectively (**Figure 5G**). When treated with Huh7-miR-183-5p inhibitor/EVs or Hep3B-miR-183-5p mimics/EVs, the SIK1 expression was increased and reduced respectively compared to NC/EVs group and the activation of PI3K/AKT pathway was negatively related to SIK1 expression level (**Figure 5H**).

Besides, to ascertain whether the effects of miR-183-5p in HCC cell-derived EVs on HUVECs are dependent on SIK1, rescue experiments were conducted. We found that overexpression of SIK1 could partially inhibit the activation of miR-183-5p on the PI3K/AKT pathway (**Figure 5I**). Similarly, knocking down SIK1 could also diminish the effect of miR-183-5p inhibitor on HUVECs (**Figure 5I**). Besides, overexpression of SIK1 inhibits the migration (**Supplementary Figure S6A**), angiogenesis (**Supplementary Figure S6B**) and permeability (**Supplementary Figure S6C**) capacity of HUVECs induced by miR-183-5p in HCC cell-derived EVs. These results revealed that miR-183-5p in HCC cell-derived EVs promoted angiogenesis capacity by inhibiting the expression of SIK1 and activating the PI3K/AKT pathway in HUVECs.

### HUVECs treated by HCC cell-derived EVs miR-183-5p promoted HCC progression in return

**Figure 6:**
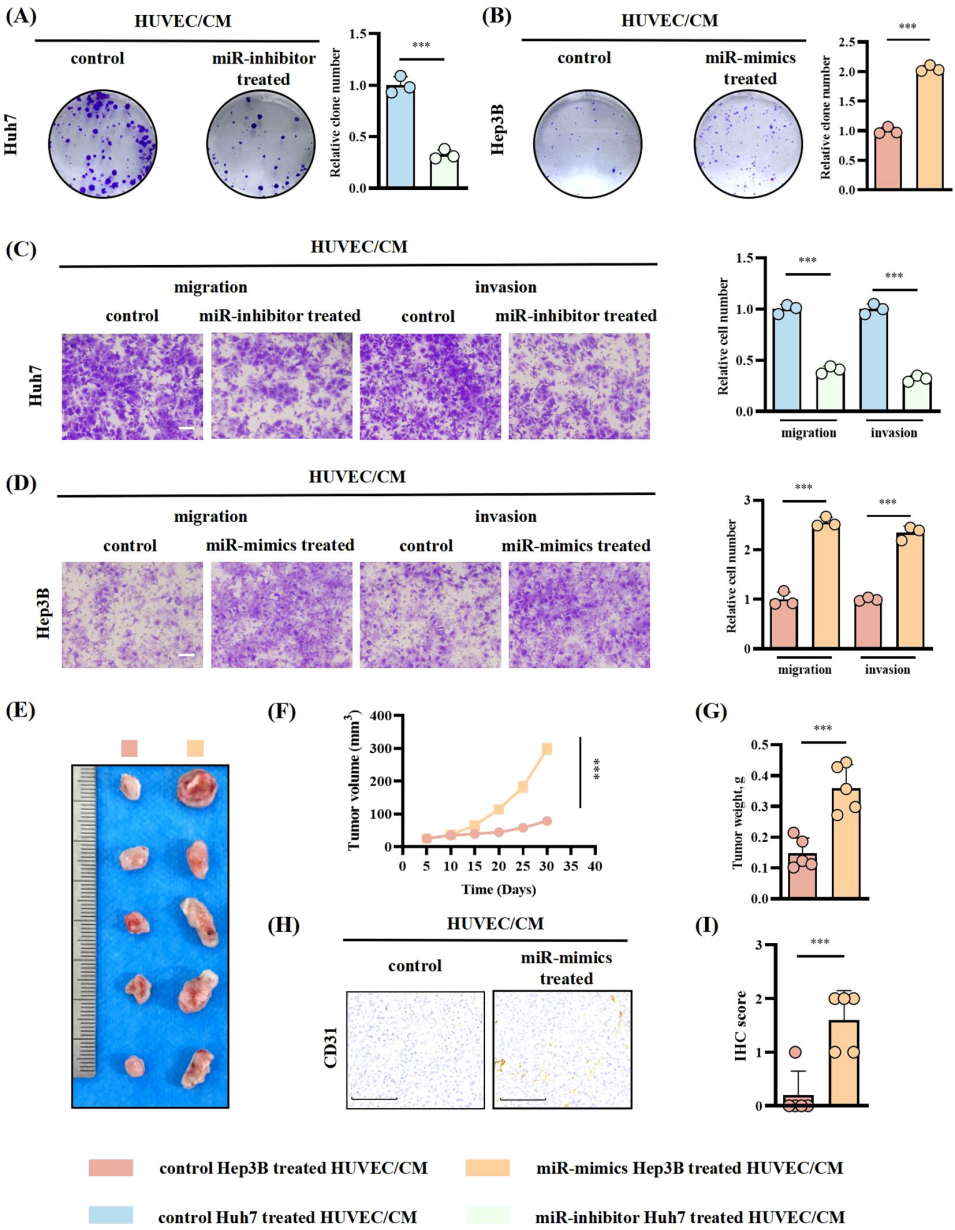
HUVECs treated with miR-183-5p-containing EVs derived from HCC cells promoted HCC progression in return. HUVECs were treated with Huh7-miR-183-5p inhibitor/EVs and Hep3B-miR-183-5p mimics/EVs, respectively, and then HUVECs/CM was incubated with Huh7/Hep3B cells. **(A, B)** Colony formation assay indicated the change in proliferation ability of Huh7 and Hep3B. Data are presented as means ± SD (n = 3). **(C, D)** Transwell assay indicated the change in migration and invasion abilities of Huh7 and Hep3B. Data are presented as means ± SD (n = 3). Scale bar: 500μm. Hep3B was cocultured with the CM of HUVECs pretreated with control or miR-183-5p mimics/EVs and then used to perform tumor xenograft model. Tumor volumes **(E, F)** and weights **(G)** were measured in the xenograft model. Data are presented as means ± SD (n = 5 in each group). **(H, I)** Relative expression level and representative IHC staining images of CD31 for indicated tumor tissues. Data are presented as means ± SD (n = 5 in each group). Scale bar: 100μm. Statistical data presents in this figure show means ± SD of three times of independent experiments. ***P < 0.001.

Considering HUVECs’ capacity to secrete chemokines and impact tumor progression through remodeling the TME (19, 20), we sought to investigate whether HUVECs/CM could exert an effect on HCC cells in return. HUVECs were treated with Huh7-miR-183-5p inhibitor/EVs and Hep3B-miR-183-5p mimics/EVs, respectively, and then HUVECs/CM was incubated with Huh7/Hep3B cells. The results showed that compared to control group, the HUVECs/CM pretreated with Hep3B-miR-183-5p mimics/EVs could promote the proliferation (**Figures 6A, B**), migration and invasion (**Figures 6C, D**) of HCC cells *in vitro*, whereas the HUVECs/CM pretreated with Huh7-miR-183-5p inhibitor/EVs exerted contrary effect, consistent with our hypothesis. *In vivo*, Hep3B was co-cultured with the HUVECs/CM pretreated with control or miR-183-5p mimics/EVs and then used to perform subcutaneous tumor model. The tumor volume (**Figures 6E, F**), weight (**Figure 6G**), and level of CD31 expression (**Figures 6H, I**) were increased for Hep3B cocultured with HUVECs/CM which were pretreated by miR-183-5p mimics/EVs.

### HUVECs treated with miR-183-5p-containing EVs derived from HCC cells promote HCC progression by activating the CCL20/CCR6 axis in HCC cells

**Figure 7:**
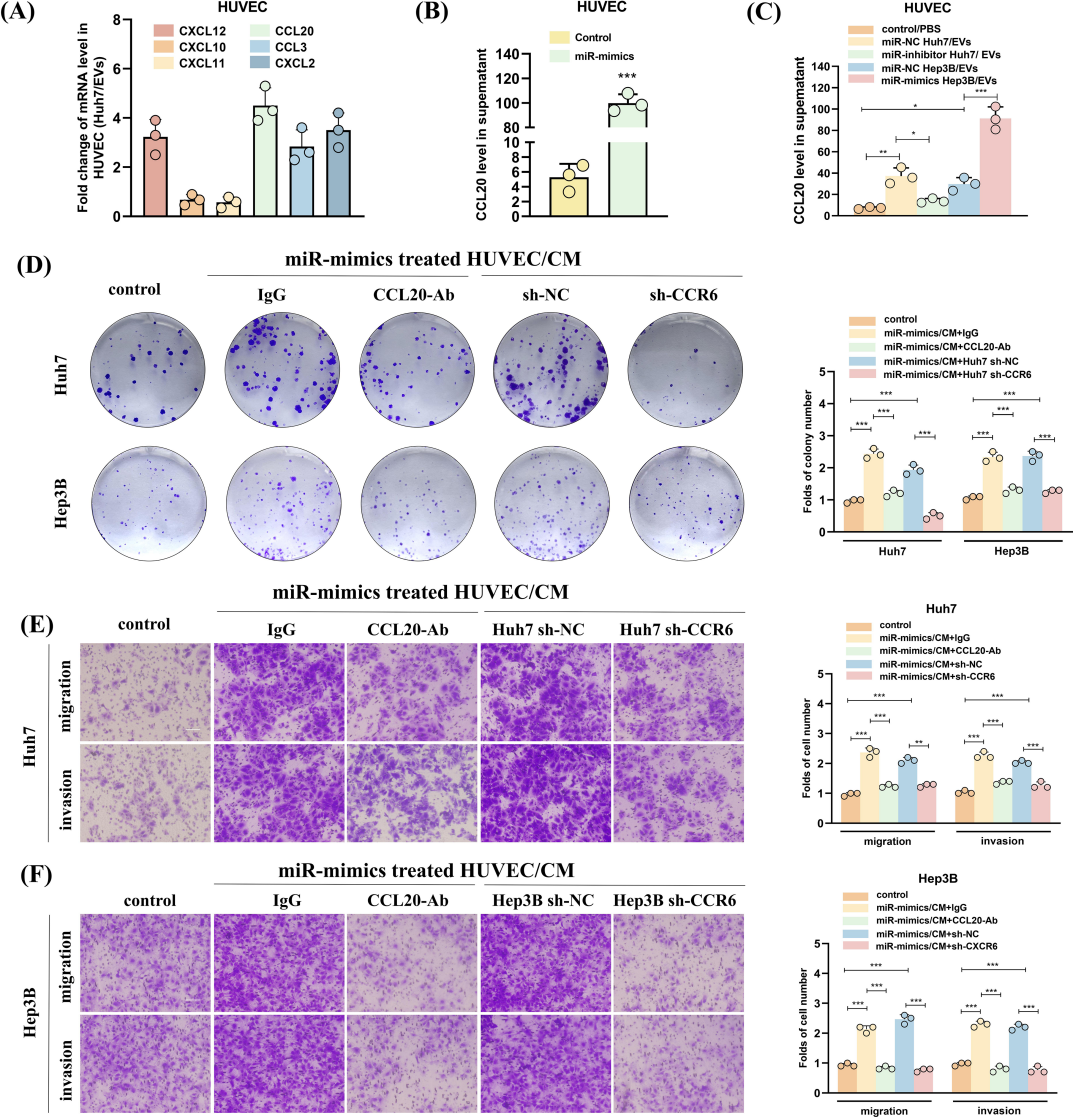
HUVECs treated with miR-183-5p-containing EVs derived from HCC cells promote HCC progression by activating the CCL20/CCR6 axis in HCC cells. **(A)** The levels of angiogenesis-associated chemokines in HUVECs treated with Huh7-derived EVs. Data are presented as mean ± SD (n = 3). **(B)** The effect of miR-183-5p on the secretion of CCL20 by HUVECs was detected using ELISA. Data are presented as means ± SD (n = 3). **(C)** The level of secretion of CCL20 by HUVECs co-cultured with HCC cell-derived EVs transfected with Hep3B-miR-183-5p mimics/NC or Huh7-miR-183-5p inhibitor/NC was determined by ELISA. Data are presented as means ± SD (n = 3). The HUVECs were co-transfected with miR-183-5p mimics, and then CCL20Ab/IgG were added. The CM was obtained and co-cultured with Huh7 and Hep3B cell lines. In addition, the CM of HUVEC cells transfected with miR-183-5p mimics was obtained, and Huh7 and Hep3B cell lines transfected with sh-CCR6/NC were cultured with CM, then **(D)** Colony formation assay indicated the change in cell proliferation ability of Huh7 and Hep3B, and **(E, F)** Transwell assay indicated the change in cell migration and invasion abilities of Huh7 and Hep3B. Data are presented as means ± SD (n = 3). Scale bar: 100μm. Statistical data presents in this figure show means ± SD of three times of independent experiments. *P < 0.05, **P < 0.01, ***P < 0.001.

Then, we assessed the levels of chemokines in HUVECs treated with Huh7 cell-derived EVs. Our findings revealed that CXCL12, CCL20, CCL3, and CXCL2 exhibited higher expression levels than the other chemokines (**Figure 7A**). Subsequent ELISA analysis showed that the secretion of CCL20 significantly increased after the transfection of miR-183-5p mimics into HUVECs (**Figure 7B**). Furthermore, treating HUVECs with Hep3B-miR-183-5p mimics/EVs significantly upregulated CCL20 expression compared with the negative control group. Conversely, the Huh7-miR-183-5p inhibitor/EVs notably decreased CCL20 expression within HUVECs (**Figure 7C**). These results suggested that CCL20 derived from HUVECs may be influential in promoting tumor progression within TME. The CCR6 is a chemokine receptor with high affinity and specificity to CCL20 (21–23), highly expressed in HCC tissues. Notably, CCL20-Ab/IgG was added to the CM from HUVECs transfected with miR-183-5p mimics, and the CM was incubated with Huh7/Hep3B cells. The colony formation and transwell assays indicated that adding CCL20-Ab prevented HCC cells proliferation, migration and invasion (**Figures 7D–F**). Furthermore, CCR6 knockdown HCC cells were co-cultured with CM from HUVECs transfected with miR-183-5p mimics. In contrast to the negative control group, CCR6 knockdown notably weakened the promotion effect of supernatant from HUVECs on proliferation, migration and invasion of HCC cells (**Figures 7D–F**).

**Figure 8:**
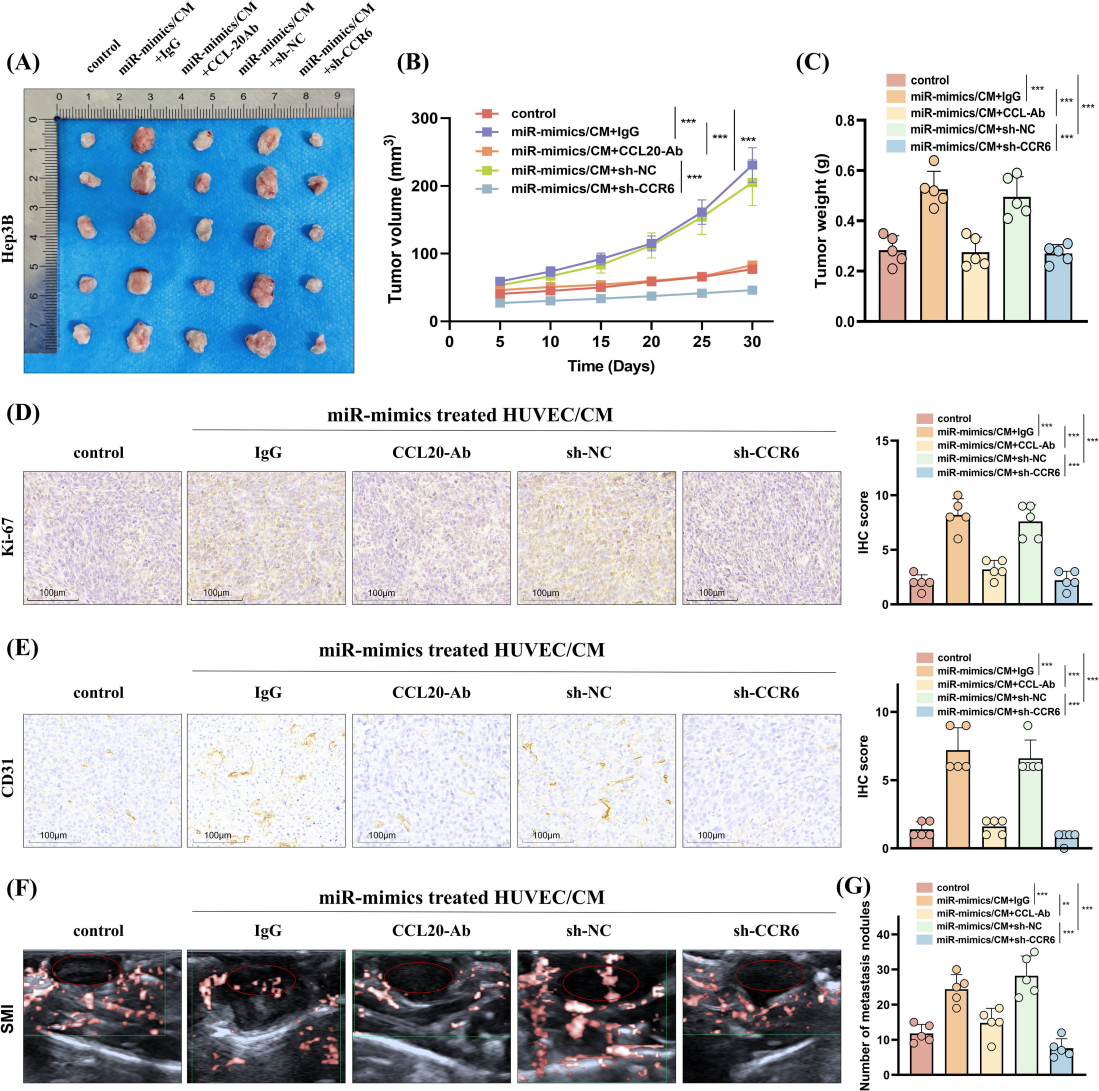
HUVECs induced by miR-183-5p delivered by EVs from HCC cells promotes HCC progression *in vivo* by activating the CCL20/CCR6 axis in HCC cells. **(A, B)** The volumes of subcutaneous tumors were measured at 5-day intervals, and growth curves were graphed. Data are presented as means ± SD (n = 5 in each group). **(C)** Additionally, tumor weights were assessed on day 30. Data are presented as means ± SD (n = 5 in each group). **(D, E)** Representative IHC staining images of Ki-67 and CD31 for subcutaneous tumor tissues. Scale bar: 100μm. Data are presented as means ± SD (n = 5 in each group). **(F)** Representative superb microvascular imaging (SMI) technique shows blood perfusion inside the tumor (n = 5 in each group). **(G)** The number of lung metastasis nodules was counted and analyzed. Data are presented as means ± SD (n = 5 in each group). **P < 0.01, ***P < 0.001.

Moreover, *in vivo* experiments evaluating subcutaneous tumor formation and lung metastasis demonstrated that treating HCC cells with CCL20-Ab or CCR6 knockdown can suppress the enhanced proliferative capacity (**Figures 8A–D**) and angiogenesis (**Figures 8E, F**) and increased number of lung metastases (**Figure 8G**, **Supplementary Figure S7**) induced by CM from HUVEC transfected with miR-183-5p mimics.

### Discussion

The occurrence and progression of malignant tumors necessitate an intricate interplay between vascular endothelial and tumor cells within the TME (24, 25). Tumor neovascularization profoundly impacts the TME, as the newly formed immature blood vessels exhibit compromised tight junctions and heightened permeability, thereby facilitating tumor advancement and metastasis (26). The molecular and signaling mechanisms governing the interaction between tumors and vascular endothelial cells are intricate. This study observed a high expression of miR-183-5p in HCC tissues, which was significantly associated with an unfavorable prognosis in patients with HCC. Importantly, we elucidated how HCC cell-derived EVs enriched with miR-183-5p facilitated communication between vascular endothelial cells and HCC cells in the TME, promoting HCC proliferation and metastasis.

Recently, numerous studies have demonstrated the ability of tumor-derived EVs to transfer highly enriched miRNAs to recipient cells, thereby inducing the inhibition of their target genes. Jiang et al. reported that EVs derived from osteosarcoma cells inhibited osteosarcoma progression by suppressing ZEB1 expression through miR-144-3p (27). Similarly, Sun et al. found that EVs-derived miR-335-5p promoted colorectal cancer progression by targeting RASA1 (28). Several studies have reported positive role of miR-183-5p in breast cancer (29), prostate cancer (30), liver cancer (31), and other tumor tissues. The reported literature showed that there are several regulatory factors that can upregulate miR-183 expression level. They can promote the transcriptional expression of miR-183 by binding to the upstream promoter region. In HCC, the regulation of Wnt/beta-catenin (CTNNB1) signaling pathway on miR-183 is the most studied (32, 33). However, few studies have focused on elucidating the molecular mechanism underlying miRNAs in HCC cell-derived EVs in the TME, particularly their crosstalk with vascular endothelial cells. Our study was the first to establish correlations between miR-183-5p plasma EVs in patients with HCC and tumor progression using CEUS techniques, which were further validated in *in vivo* and *in vitro* experiments. Collectively, our findings provide compelling evidence supporting the miR-183-5p role in HCC cell-derived EVs in promoting tumor progression. The miRNA content of EVs in peripheral blood is the most transformative molecular biomarker for liquid biopsy in cancer (34). Our study found that the expression of miR-183-5p/EVs in plasma samples from patients with HCC was significantly elevated compared with that in plasma samples from the normal control population, making it a highly transformative molecular indicator for liquid biopsy in cancer research. Additionally, the levels of miR-183-5p/EVs in plasma were higher in low-grade patients with HCC than in high-grade patients with HCC, suggesting its potential as a valuable molecular marker for both HCC diagnosis and prognosis assessment. These findings contribute to advancing liquid biopsy techniques and hold promise for future developments. Liquid biopsy is a technique for early diagnosis of cancer by collecting body fluid samples such as blood and urine and detecting tumor markers in them. Compared to traditional tissue biopsies, liquid biopsies are less invasive and more convenient, and because of this, they have developed into an emerging field in tumor diagnosis and prognosis assessment. The results of our study show that the miR-183-5p level of EVs in plasma is significantly elevated, suggesting that it can be used not only as a potential diagnostic marker, but also to aid in assessing tumor progression. Further analysis also found that miR-183-5p levels in EVs were significantly higher in patients with low-grade HCC, suggesting that miR-183-5p is not only closely related to tumor progression, but also has the potential to serve as an important molecular marker for evaluating HCC grading. This finding provides a new research direction for the early diagnosis, staging assessment and individualized treatment of HCC.

Furthermore, TME encompasses the internal and external surroundings in which tumor growth occurs, comprising a diverse array of immune cells, endothelial cells, and tumor-associated fibroblasts (35). Recently, EVs have been discovered within the TME, with mounting evidence suggesting that these EVs carry genes responsible for regulating metastasis and angiogenesis. Within the TME context, EVs facilitate the transfer of functional biomolecules, such as miRNAs, to target cells to promote tumor progression by regulating angiogenesis (36, 37). This study demonstrated that miR-183-5p from HCC cell-derived EVs metastasizes into HUVECs. Furthermore, miR-183-5p from HCC cell-derived EVs downregulated SIK1 expression while activating the PI3K/AKT signaling pathway in HUVECs to induce neovascularization. The small protein CCL20, called liver activation-regulated chemokine, is naturally produced in the liver, colon, and skin. It is crucial in tissue inflammation and balance and binds to the receptor CCR6. Recently, it has been reported that the CCL20/CCR6 axis is implicated in HCC progression (38), colorectal cancer (39) and pancreatic cancer (40). Wang et al. also reported that the CCL20/CCR6 axis can promote lung metastasis of HCC (21). In this study, we demonstrated that miR-183-5p derived from HCC cell-derived EVs induces endothelial cell angiogenesis, facilitating crosstalk between tumor and endothelial cells through the CCL20/CCR6 axis, thereby promoting HCC progression. EVs-miR-183-5p, as a biomarker, can reflect the biological characteristics of HCC cells, providing new ideas for early detection, optimal management and treatment of HCC.

The limitation of this study is that we did not investigate the upstream mechanism of EVs-miR-183-5p in depth. In the future, we will conduct experiments with more rigorous scientific design to explore upstream mechanisms leading to upregulation of miR-183-5p expression. Besides, whether the SIK1/PI3K/AKT and CCL20/CCR6 signaling pathways for miR-183-5p are specific in HCC and whether the axis has an effect on angiogenesis in other cancers remain questions that need to be explored.

## Conclusions

**Figure 9:**
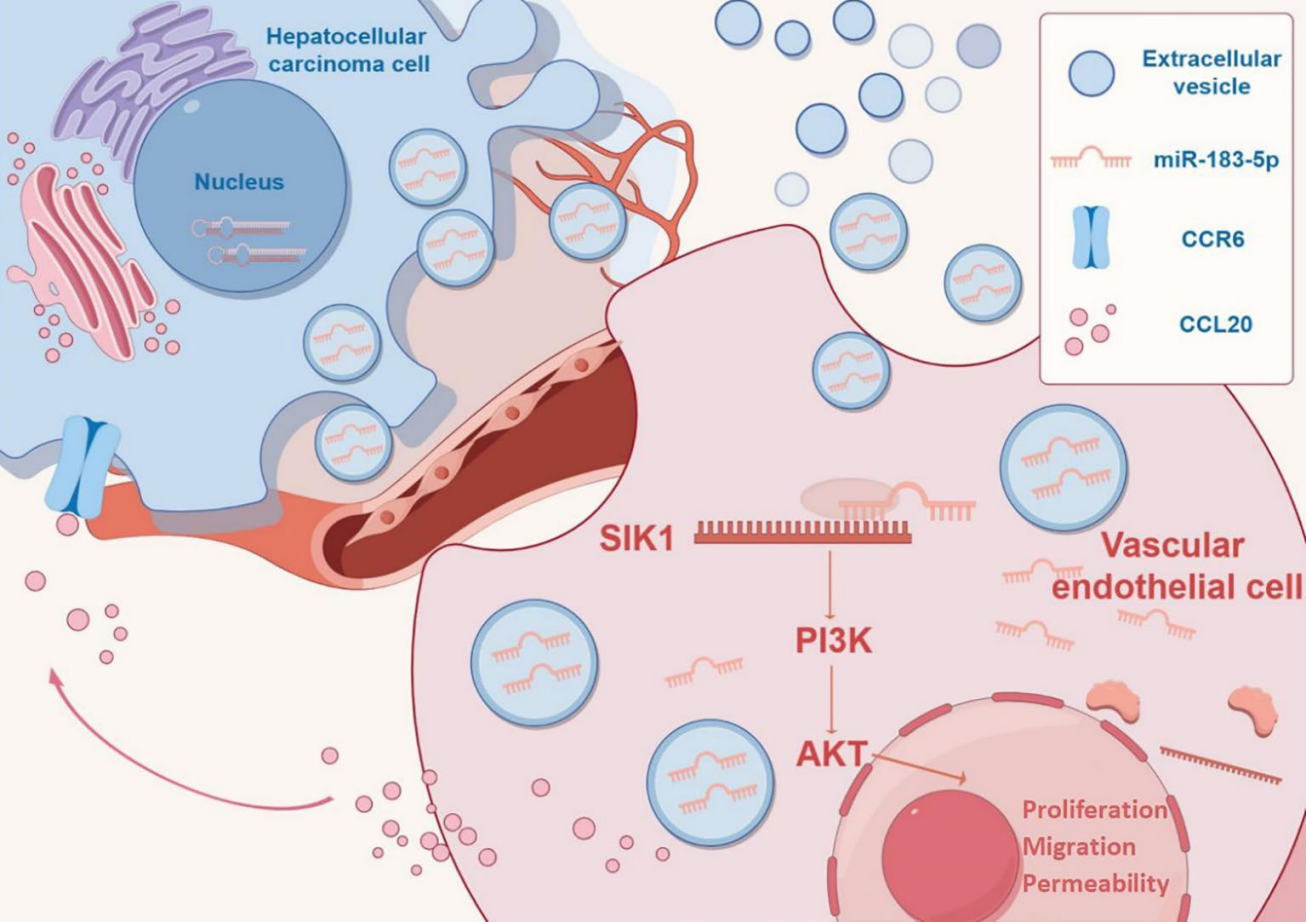
Mechanism diagram by Figdraw (https://www.figdraw.com).

In conclusion, we demonstrated that miR-183-5p in HCC cell-derived EVs induces proliferation, migration, angiogenesis and permeability in HUVECs by down-regulating SIK1 expression and activating the PI3K/AKT signaling pathway. Furthermore, we found that stimulated HUVECs could secrete the chemokine CCL20 and induce HCC progression through the CCL20/CCR6 signal pathway in return, thereby facilitating HCC progression (**Figure 9**). Therefore, targeting the intercellular communication mediated by miR-183-5p enriched in HCC cell-derived EVs might be a potential therapeutic target for HCC.

## Data Availability

The datasets presented in this study can be found in online repositories. The names of the repository/repositories and accession number(s) can be found in the article/**Supplementary Material**.

## Supplementary material

Supplementary Figure 1 The expression of miR-183-5p in EVs derived from five cell lines. Data are presented as means ± SD (n = 3).

Supplementary Figure 2
**(A, B)** CCK-8 assay for HUVECs treated by Huh7/EVs and Hep3B/EVs with or without GW4869. Data are presented as means ± SD (n = 3).

Supplementary Figure 3
**(A, B)** miR-183-5p mimics and miR-183-5p inhibitor respectively loaded into Hep3B and Huh7 cells and the EVs released by them. Data are presented as means ± SD (n = 3).

Supplementary Figure 4
**(A, B)** The proliferation capacity of HUVECs treated with miR-183-5p mimics Hep3B/EVs and miR-183-5p inhibitors Huh7/EVs was detected by CCK-8 assay. Data are presented as mean ± SD (n = 3).

Supplementary Figure 5
**(A)** Knocking down SIK1 could promoted ROS generation in HUVECs. **(B)** Overexpression of SIK1 could inhibit ROS generation in HUVECs. Scale bar: 100μm.

Supplementary Figure 6
**(A–C)** Overexpression of SIK1 inhibits the migration, angiogenesis and permeability capacity of HUVECs induced by miR-183-5p in HCC cell-derived EVs. **(A)** Data are presented as means ± SD (n = 3). Scale bar: 25μm. **(B)** Data are presented as means ± SD (n = 3). Scale bar: 50μm. **(C)** Data are presented as means ± SD (n = 3). Scale bar: 25μm.

Supplementary Figure 7 Representative H&E staining of mice lung sections showing tumor lesions. Scale bar: 500μm.

Supplementary Table 1 The primer sequences of qRT-PCR.

Supplementary Table 2 Sequences of shRNA for SIK1.

Supplementary Table 3 Sequences of shRNA for CCR6.

Supplementary Table 4 Sequences of miR-183-5p mimics and negative control.

Supplementary Table 5 Sequences of miR-183-5p inhibitor and negative control.

Supplementary Table 6 The information of antibodies.

## References

[1] Freddie B Mathieu L Hyuna S Jacques F Rebecca LS Isabelle S. Global cancer statistics 2022: GLOBOCAN estimates of incidence and mortality worldwide for 36 cancers in 185 countries. CA Cancer J Clin. (2024) 74(3):229–63. doi: 10.3322/caac.21834 38572751

[2] Abhange K Makler A Wen Y Ramnauth N Mao W Asghar W. Small extracellular vesicles in cancer. Bioactive materials. (2021) 6:3705–43. doi: 10.1016/j.bioactmat.2021.03.015 PMC8056276 33898874

[3] Wang X Huang H Sze K Wang J Tian L Lu J. S100A10 promotes HCC development and progression via transfer in extracellular vesicles and regulating their protein cargos. Gut. (2023) 72:1370–84. doi: 10.1136/gutjnl-2022-327998 PMC10314046 36631249

[4] Fang T Lv H Lv G Li T Wang C Han Q. Tumor-derived exosomal miR-1247-3p induces cancer-associated fibroblast activation to foster lung metastasis of liver cancer. Nat Commun. (2018) 9:191. doi: 10.1038/s41467-017-02583-0 29335551 PMC5768693

[5] Zhou Y Ren H Dai B Li J Shang L Huang J. Hepatocellular carcinoma-derived exosomal miRNA-21 contributes to tumor progression by converting hepatocyte stellate cells to cancer-associated fibroblasts. J Exp Clin Cancer research: CR. (2018) 37:324. doi: 10.1186/s13046-018-0965-2 30591064 PMC6307162

[6] Zhou J Che J Xu L Yang W Zhou W Zhou C. Tumor-derived extracellular vesicles containing long noncoding RNA PART1 exert oncogenic effect in hepatocellular carcinoma by polarizing macrophages into M2. Digestive liver disease: Off J Ital Soc Gastroenterol Ital Assoc Study Liver. (2022) 54:543–53. doi: 10.1016/j.dld.2021.07.005 34497040

[7] WeiHong R XuRan Z WenBo L Qian F HuiJie F Yan T. Exosomal miRNA-107 induces myeloid-derived suppressor cell expansion in gastric cancer. Cancer Manag Res. (2019) 11:4023–40. doi: 10.2147/CMAR.S198886 PMC6511657 31190980

[8] Fang JH Zhang ZJ Shang LR Luo YW Lin YF Yuan Y. Hepatoma cell-secreted exosomal microRNA-103 increases vascular permeability and promotes metastasis by targeting junction proteins. Hepatol (Baltimore Md). (2018) 68:1459–75. doi: 10.1002/hep.29920 29637568

[9] Zeng Z Li Y Pan Y Lan X Song F Sun J. Cancer-derived exosomal miR-25-3p promotes pre-metastatic niche formation by inducing vascular permeability and angiogenesis. Nat Commun. (2018) 9:5395. doi: 10.1038/s41467-018-07810-w 30568162 PMC6300604

[10] Wang H Wei H Wang J Li L Chen A Li Z. MicroRNA-181d-5p-containing exosomes derived from CAFs promote EMT by regulating CDX2/HOXA5 in breast cancer. Mol Ther Nucleic Acids. (2020) 19:654–67. doi: 10.1016/j.omtn.2019.11.024 PMC6970169 31955007

[11] Wei K Ma Z Yang F Zhao X Jiang W Pan C. M2 macrophage-derived exosomes promote lung adenocarcinoma progression by delivering miR-942. Cancer letters. (2022) 526:205–16. doi: 10.1016/j.canlet.2021.10.045 34838826

[12] Lu Y Han G Zhang Y Zhang L Li Z Wang Q. M2 macrophage-secreted exosomes promote metastasis and increase vascular permeability in hepatocellular carcinoma. Cell communication signaling: CCS. (2023) 21:299. doi: 10.1186/s12964-022-00872-w 37904170 PMC10614338

[13] Yong Z Fubo Z Xiaoling Y Fei L Fangyi L Ping L. Quantitative dynamic contrast-enhanced ultrasound may help predict the outcome of hepatocellular carcinoma after microwave ablation. Int J Hyperthermia. (2018) 35(1):105–11. doi: 10.1080/02656736.2018.1483533 30300039

[14] Jin Woo C Jung Hoon K Hyo-Cheol K Won Seok C Song Yi B Kyoungbun L. Comparison of tumor vascularity and hemodynamics in three rat hepatoma models. Abdom Radiol (NY). (2016) 41(2):257–64. doi: 10.1007/s00261-015-0591-9 26867907

[15] Hui T Qi W. Quantitative analysis of microcirculation blood perfusion in patients with hepatocellular carcinoma before and after transcatheter arterial chemoembolisation using contrast-enhanced ultrasound. Eur J Cancer. (2016) 68:82–9. doi: 10.1016/j.ejca.2016.08.016 27728840

[16] Zicheng S Qiwei J Bing G Xiaomei Z Lang B Lei W. AKT blocks SIK1-mediated repression of STAT3 to promote breast tumorigenesis. Cancer Res. (2023) 83(8):1264–79. doi: 10.1158/0008-5472.CAN-22-3407 36806887

[17] Bo K Xin-Di W Xiao-Peng S Qin Q Ming Y Yan-Ning Y. LKB1 inhibits proliferation, metastasis and angiogenesis of thyroid cancer by upregulating SIK1. J Cancer. (2022) 13(9):2872–83. doi: 10.7150/jca.72021 PMC9330453 35912012

[18] Xueyi C Ping L Wei Z Xiaofang L Caihua W Feifei H. ETNPPL modulates hyperinsulinemia-induced insulin resistance through the SIK1/ROS-mediated inactivation of the PI3K/AKT signaling pathway in hepatocytes. J Cell Physiol. (2023) 238(5):1046–62. doi: 10.1002/jcp.30993 36924049

[19] Pedro B Sandra Isabel A Frederico A-D-S Joana Nunes Ribeiro D. Chemokine-directed tumor microenvironment modulation in cancer immunotherapy. Int J Mol Sci. (2021) 22(18):9804. doi: 10.3390/ijms22189804 34575965 PMC8464715

[20] Nisha N Max SW Weiping Z. Chemokines in the cancer microenvironment and their relevance in cancer immunotherapy. Nat Rev Immunol. (2017) 17(9):559–72. doi: 10.1038/nri.2017.49 PMC5731833 28555670

[21] Wang L Qiao C Han L Wang X Miao J Cao L. HOXD3 promotes the migration and angiogenesis of hepatocellular carcinoma via modifying hepatocellular carcinoma cells exosome-delivered CCR6 and regulating chromatin conformation of CCL20. Cell Death Dis. (2024) 15:221. doi: 10.1038/s41419-024-06593-x 38493218 PMC10944507

[22] Suguru K Kouji I Atsushi M. The CCL20-CCR6 axis in cancer progression. Int J Mol Sci. (2020) 21(15):5186. doi: 10.3390/ijms21155186 32707869 PMC7432448

[23] Vilma Oliveira F Claudia R Ulrich K Pirus G. Chemokine/chemokine receptor pair CCL20/CCR6 in human colorectal Malignancy: An overview. World J Gastroenterol. (2016) 22(2):833–41. doi: 10.3748/wjg.v22.i2.833 PMC4716081 26811629

[24] Liren Z Jiali X Suiqing Z Feifan Y Ruizhi Z Wenhua Y. Endothelial DGKG promotes tumor angiogenesis and immune evasion in hepatocellular carcinoma. J Hepatol. (2023) 80(1):82–98. doi: 10.1016/j.jhep.2023.10.006 37838036

[25] Roberta L Mohanraj R Anna D. Tumor angiogenesis: causes, consequences, challenges and opportunities. Cell Mol Life Sci. (2019) 77(9):1745–70. doi: 10.1007/s00018-019-03351-7 PMC7190605 31690961

[26] Wangjie J Xiaoli S Lizhu S Yaodong Z Xiangxu K Xiao Y. Exosomal miR-30a-5p promoted intrahepatic cholangiocarcinoma progression by increasing angiogenesis and vascular permeability in PDCD10 dependent manner. Int J Biol Sci. (2023) 19(14):4571–87. doi: 10.7150/ijbs.83170 PMC10535699 37781039

[27] Mingyang J Yiji J Kaicheng L Fu G Ke Z Mingjing X. Exosome-mediated miR-144-3p promotes ferroptosis to inhibit osteosarcoma proliferation, migration, and invasion through regulating ZEB1. Mol Cancer. (2023) 22(1):113. doi: 10.1186/s12943-023-01804-z 37461104 PMC10351131

[28] Xuecheng S Feiyan L Wenjing S Weijian Z Daoquan F Lifang L. Exosome-transmitted miRNA-335-5p promotes colorectal cancer invasion and metastasis by facilitating EMT via targeting RASA1. Mol Ther Nucleic Acids. (2021) 24:164–74. doi: 10.1016/j.omtn.2021.02.022 PMC7960496 33767913

[29] Masahisa K Nicole Z Rosario D Giovanni N Dario V Pierluigi G. Synergistic apoptotic effect of miR-183-5p and Polo-Like kinase 1 inhibitor NMS-P937 in breast cancer cells. Cell Death Differ. (2021) 29(2):407–19. doi: 10.1038/s41418-021-00864-2 PMC8816952 34561554

[30] Lin J Yue L Ying-Chun Z Hai T. MiR-183-5p promotes tumor progression of osteosarcoma and predicts poor prognosis in patients. Cancer Manag Res. (2021) 13:805–14. doi: 10.2147/CMAR.S285909 PMC7850385 33536788

[31] Marks MI Shapera RM Brazeau M. Pediatric antimicrobial therapy. VI. Can Med Assoc J. (1973) 109. PMC1946908 4742920

[32] Leung W He M Chan A Law P Wong N. Wnt/β-Catenin activates MiR-183/96/182 expression in hepatocellular carcinoma that promotes cell invasion. Cancer Lett. (2015) 362:97–105. doi: 10.1016/j.canlet.2015.03.023 25813403

[33] Nie X Liu Y Chen W Wang Y. Interplay of miRNAs and canonical wnt signaling pathway in hepatocellular carcinoma. Front Pharmacol. (2018) 9:657. doi: 10.3389/fphar.2018.00657 29977206 PMC6021530

[34] Karama A Valerie D Catherine T Michelle D Marie-Ange D-P Rodney JO. Extracellular vesicle-based liquid biopsy biomarkers and their application in precision immuno-oncology. biomark Res. (2023) 11(1):99. doi: 10.1186/s40364-023-00540-2 37978566 PMC10655470

[35] Yi X Dihua Y. Tumor microenvironment as a therapeutic target in cancer. Pharmacol Ther. (2020) 221:107753. doi: 10.1016/j.pharmthera.2020.107753 33259885 PMC8084948

[36] Shunsuke K Kazuyuki S Mika T Akihiko S Tomohiro A Tomoya H. Exosomes secreted from cancer-associated fibroblasts elicit anti-pyrimidine drug resistance through modulation of its transporter in Malignant lymphoma. Oncogene. (2021) 40(23):3989–4003. doi: 10.1038/s41388-021-01829-y PMC8195743 33994542

[37] Ming-Zhu J Wei-Lin J. The updated landscape of tumor microenvironment and drug repurposing. Signal Transduct Target Ther. (2020) 5(1):166. doi: 10.1038/s41392-020-00280-x 32843638 PMC7447642

[38] Hongwu T Shuyun W Ludong Z. A tumour-promoting role of Th9 cells in hepatocellular carcinoma through CCL20 and STAT3 pathways. Clin Exp Pharmacol Physiol. (2016) 44(2):213–21. doi: 10.1111/1440-1681.12689 27797409

[39] Dan W Li Y Weina Y Qian W Jingyao L Feng L. Colorectal cancer cell-derived CCL20 recruits regulatory T cells to promote chemoresistance via FOXO1/CEBPB/NF-κB signaling. J Immunother Cancer. (2019) 7(1):215. doi: 10.1186/s40425-019-0701-2 31395078 PMC6688336

[40] Claudia G Frauke G Anita D Robert H Philip R Karen L. Role of CCL20 mediated immune cell recruitment in NF-κB mediated TRAIL resistance of pancreatic cancer. Biochim Biophys Acta Mol Cell Res. (2017) 1864(5):782–96. doi: 10.1016/j.bbamcr.2017.02.005 28188806

