# Supplementary material for: miR-183-5p-enriched extracellular vesicles promote the crosstalk between hepatocellular carcinoma cell and endothelial cell via SIK1/PI3K/AKT and CCL20/CCR6 signaling pathways
Source: Front Oncol. 2025 Mar 6;15:1532239. doi: 10.3389/fonc.2025.1532239 (PMC11922695; doi:10.3389/fonc.2025.1532239)
Supplement: Supplementary Figure 1 — The expression of miR-183-5p in EVs derived from five cell lines. Data are presented as means ± SD (n = 3). [file DataSheet1.docx]

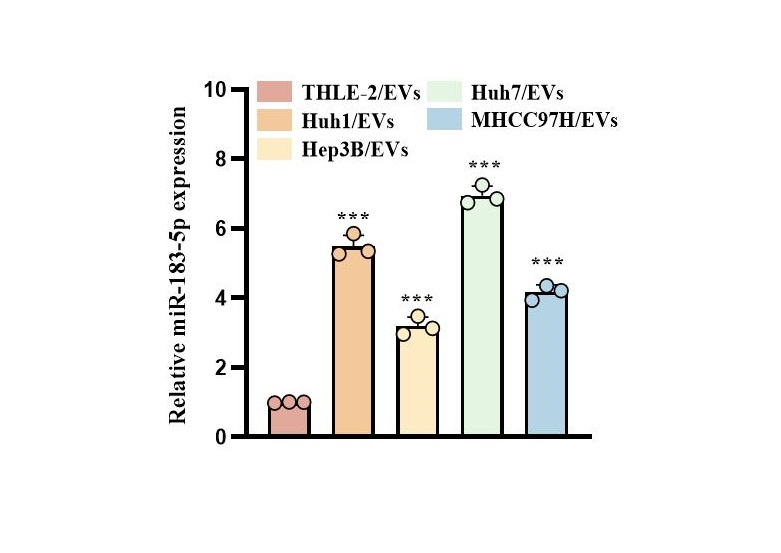


**Supplementary Figure 1**


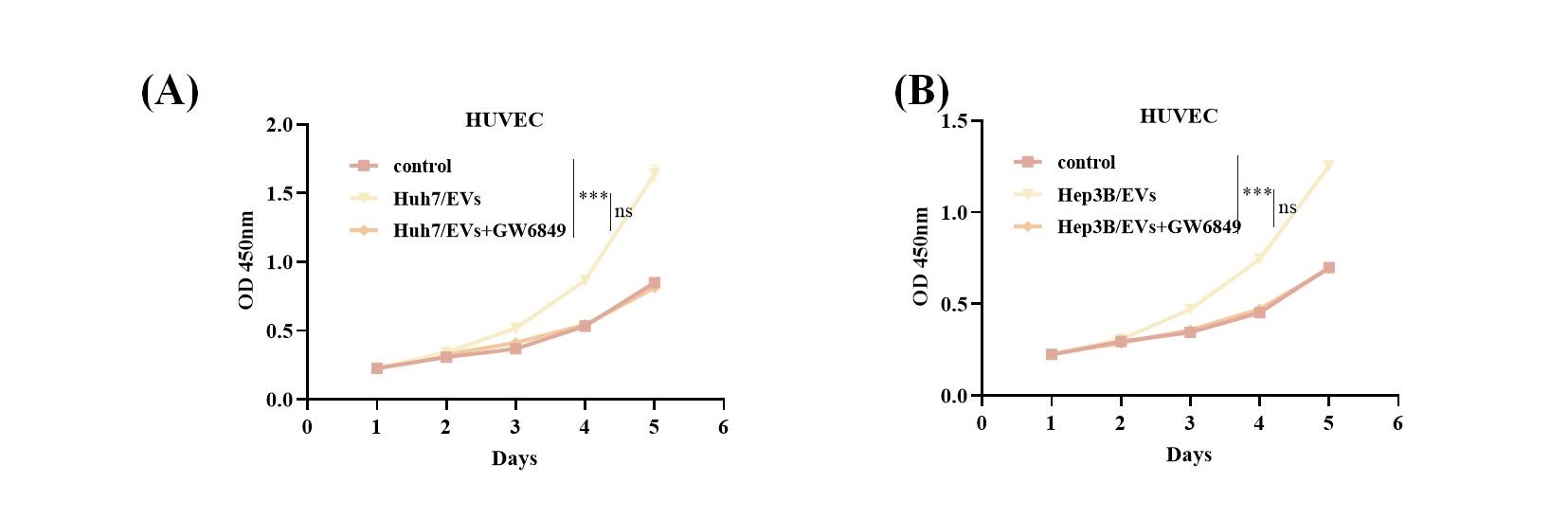


**Supplementary Figure 2**

**
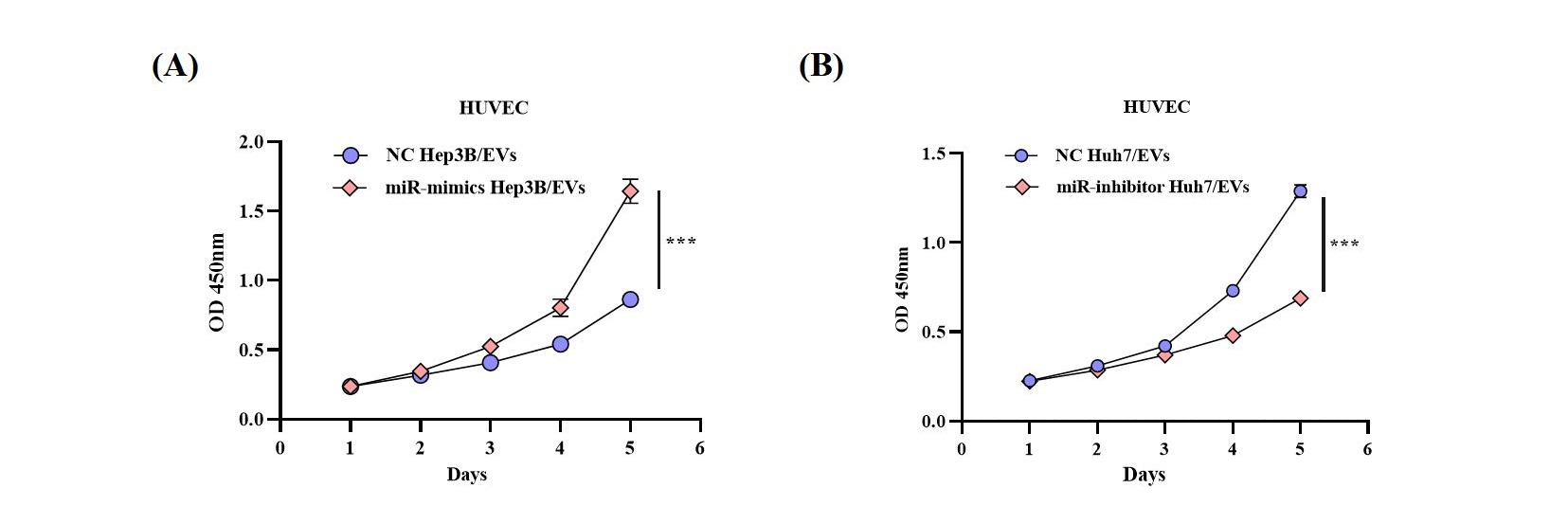

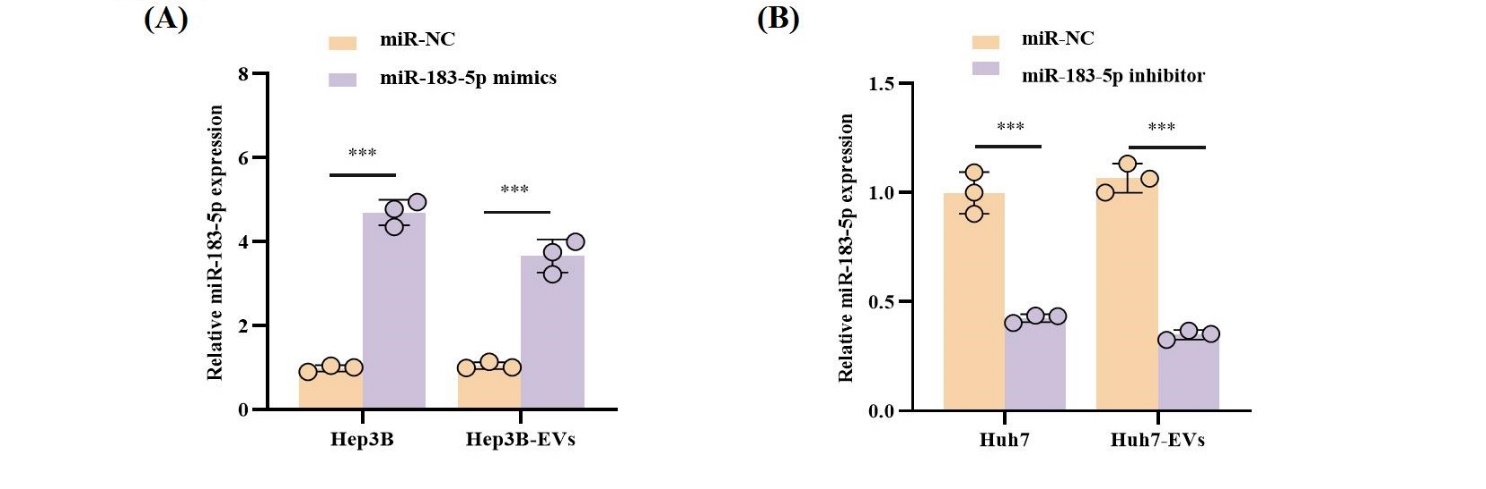
Supplementary Figure 3**


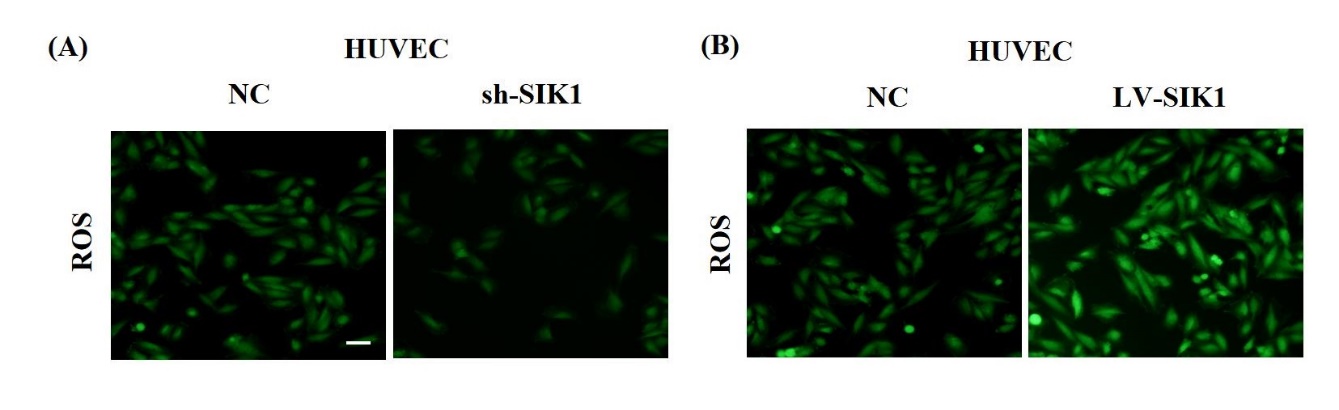
**Supplementary Figure 4**

**Supplementary Figure 5**


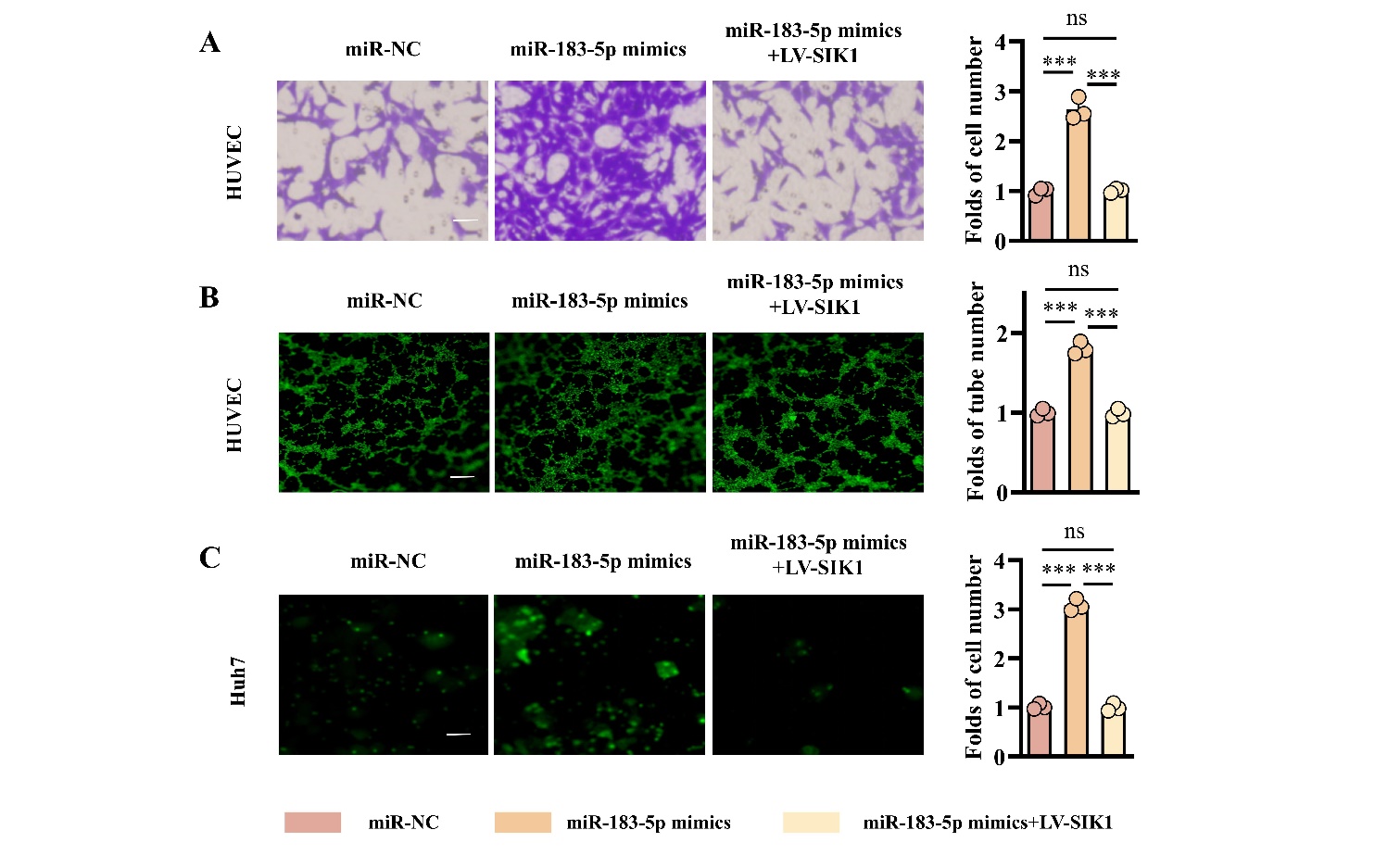
**Supplementary Figure 6**

**
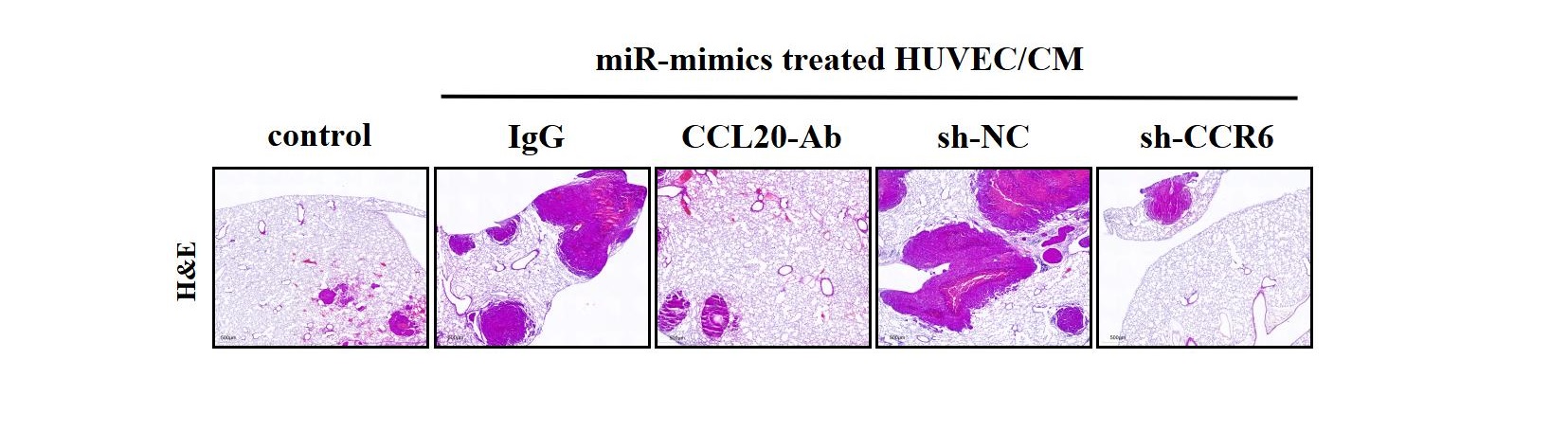
**

**Supplementary Figure 7**

| **Gene** | **Forward primer** | **Reverse primer** |
| --- | --- | --- |
| SIK1 | ACTCACCGCGCCATGTATAG | GCTTTTGCTCGCAGAGAAGG |
| miR-130b-3p | ACACTCCAGCTGGGCAGTGCAATGA | CTCAACTGGTGTCGTGGA |
| miR-532-5p | ACACTCCAGCTGGGCATGCCTTGA | CTCAACTGGTGTCGTGGA |
| miR-502-3p | ACACTCCAGCTGGGAATGCACCTGG | CTCAACTGGTGTCGTGGA |
| miR-182-5p | ACACTCCAGCTGGGTTTGGCAATGG | CTCAACTGGTGTCGTGGA |
| miR-96-5p | ACACTCCAGCTGGGTTTGGCACTAG | CTCAACTGGTGTCGTGGA |
| miR-548o-3p | ACACTCCAGCTGGGCCAAAACTGCA | CTCAACTGGTGTCGTGGA |
| miR-151a-3p | CGCGCTAGACTGAAGCTCC | AGTGCAGGGTCCGAGGTATT |
| miR-183-5p | CGCGTATGGCACTGGTAGAA | AGTGCAGGGTCCGAGGTATT |
| U6 | CTCGCTTCGGCAGCACA | AACGCTTCACGAATTTGCGT |
| GAPDH | GTCTCCTCTGACTTCAACAGCG | ACCACCCTGTTGCTGTAGCCAA |
| Cel-miR-39 | UCACCGGGUGUAAAUCAGCUUG |  |

| **Gene** | **RT primer** |
| --- | --- |
| miR-130b-3p | CTCAACTGGTGTCGTGGAGTCGGCAATTCAGTTGAGATGCCCTTTC |
| miR-532-5p | CTCAACTGGTGTCGTGGAGTCGGCAATTCAGTTGAGACGGTCCTAC |
| miR-502-3p | CTCAACTGGTGTCGTGGAGTCGGCAATTCAGTTGAGTGAATCCTTG |
| miR-182-5p | CTCAACTGGTGTCGTGGAGTCGGCAATTCAGTTGAGAGTGTGAGTT |
| miR-96-5p | CTCAACTGGTGTCGTGGAGTCGGCAATTCAGTTGAGAGCAAAAATG |
| miR-548o-3p | CTCAACTGGTGTCGTGGAGTCGGCAATTCAGTTGAGGCAAAAGTAA |
| miR-151a-3p | GTCGTATCCAGTGCAGGGTCCGAGGTATTCGCACTGGATACGACCCTCAA |
| miR-183-5p | GTCGTATCCAGTGCAGGGTCCGAGGTATTCGCACTGGATACGACAGTGAA |

**Supplementary Table 1**

| **shRNA** | **Sequence 5’---3’** |
| --- | --- |
| sh-NC | TTCTCCGAACGTGTCACGT |
| sh-SIK1 | ACGATTAGATTCAAGCAATTT |

**Supplementary Table 2**

| **shRNA** | **Sequence 5’---3’** |
| --- | --- |
| sh-NC | TTCTCCGAACGTGTCACGT |
| sh-CCR6 | TACTGGGCATCTACACTATTA |

**Supplementary Table 3**

|  | **Sequence 5’---3’** |
| --- | --- |
| miR-183-5p mimics | sense: UAUGGCACUGGUAGAAUUCACU  antisense: AGUGAAUUCUACCAGUGCCAUA |
| negative control | sense: UUCUCCGAACGUGUCACGUTT  antisense: ACGUGACACGUUCGGAGAATT |

**Supplementary Table 4**

|  | **Sequence 5’---3’** |
| --- | --- |
| miR-183-5p inhibitor | AGUGAAUUCUACCAGUGCCAUA |
| negative control | CAGUACUUUUGUGUAGUACAA |

**Supplementary Table 5**

| **Antibodies** | **SOURCE** | **IDENTIFIER** |
| --- | --- | --- |
| SIK1 | Cell Signaling Technology | #67776 |
| AKT | Cell Signaling Technology | #2920 |
| p-AKT | Proteintech | 80455-1-RR |
| PI3K | Proteintech | 60225-1-Ig |
| p-PI3K | Affinity | #AF3242 |
| β-actin | ZSGB-BIO | TA-09 |
| GAPDH | Proteintech | 60004-1-1g |
| CD81 | Abcam | ab79559 |
| CD9 | Abcam | ab236630 |
| Alix | Proteintech | 67715-1-Ig |
| Calnexin | Cell Signaling Technology | #2433S |
| GM130 | Affinity | #DF7556 |
| Ki-67 | Proteintech | 28074-1-AP |
| CD31 | Cell Signaling Technology | #77699 |

**Supplementary Table 6**
